# Association of polybrominated diphenyl ether (PBDE) levels with biomarkers of placental development and disease during mid-gestation

**DOI:** 10.1186/s12940-020-00617-7

**Published:** 2020-06-03

**Authors:** Julia R. Varshavsky, Joshua F. Robinson, Yan Zhou, Kenisha A. Puckett, Elaine Kwan, Sirirak Buarpung, Rayyan Aburajab, Stephanie L. Gaw, Saunak Sen, Sabrina Crispo Smith, Julie Frankenfield, June-Soo Park, Susan J. Fisher, Tracey J. Woodruff

**Affiliations:** 1 Program on Reproductive Health and the Environment, UCSF Department of Obstetrics, Gynecology & Reproductive Sciences, University of California, Mailstop 0132, 550 16th Street, 7th Floor, San Francisco, CA 94143 USA; 2 Center for Reproductive Sciences and Department of Obstetrics, Gynecology & Reproductive Sciences, University of California, 513 Parnassus Avenue, San Francisco, CA 94143 USA; 3 Division of Maternal-Fetal Medicine and Department of Obstetrics, Gynecology & Reproductive Sciences, University of California, 550 16th Street, 7th Floor, San Francisco, CA 94143 USA; 4 Department of Preventive Medicine, University of Tennessee Health Science Center, 66 North Pauline St, Memphis, TN 38163 USA; 5 California Environmental Protection Agency, Department of Toxic Substances Control, Environmental Chemistry Laboratory, 700 Heinz Ave # 200, Berkeley, CA 94710 USA

**Keywords:** Flame retardants, Biomonitoring, Developmental/reproductive health effects, Endocrine disruption, Preeclampsia, Pregnancy complications, Maternal health, Birth outcomes, Cytotrophoblast differentiation

## Abstract

**Background:**

Polybrominated diphenyl ether (PBDE) exposures have been associated with adverse pregnancy outcomes. A hypothesized mechanism is via alterations in placental development and function. However, we lack biomarkers that can be used as early indicators of maternal/fetal response to PBDE exposures and/or perturbations in placental development or function.

**Methods:**

To evaluate the relationship between PBDE levels and placental biomarkers during mid-gestation of human pregnancy (*n* = 62), we immunolocalized three molecules that play key roles in cytotrophoblast (CTB) differentiation and interstitial/endovascular uterine invasion—integrin alpha-1 (ITGA1*)*, vascular endothelial-cadherin (CDH5), and metalloproteinase-1 (MMP1)–and assessed three morphological parameters as potential indicators of pathological alterations using H&E-stained tissues–leukocyte infiltration, fibrinoid deposition, and CTB endovascular invasion. We evaluated associations between placental PBDE levels and of biomarkers of placental development and disease using censored Kendall’s tau correlation and linear regression methods.

**Results:**

PBDEs were detected in all placental samples. We observed substantial variation in antigen expression and morphological endpoints across placental regions. We observed an association between PBDE concentrations and immunoreactivity of endovascular CTB staining with anti-ITGA1 (inverse) or interstitial CTBs staining with anti-CDH5 (positive).

**Conclusions:**

We found several molecular markers that may be sensitive placental indicators of PBDE exposure. Further, this indicates that placental biomarkers of development and disease could be useful barometers of exposure to PBDEs, a paradigm that could be extended to other environmental chemicals and placental stage-specific antigens.

## Background

Pregnancy complications, such as preterm birth, gestational diabetes, fetal growth restriction, hypertension, and preeclampsia, are significantly linked with adverse maternal and fetal health outcomes [1], and environmental exposures are important risk factors [2–4]. Environmental chemicals can exert their influence on pregnancy through multiple mechanisms, including alterations in placental development and function.

**Fig. 1:**
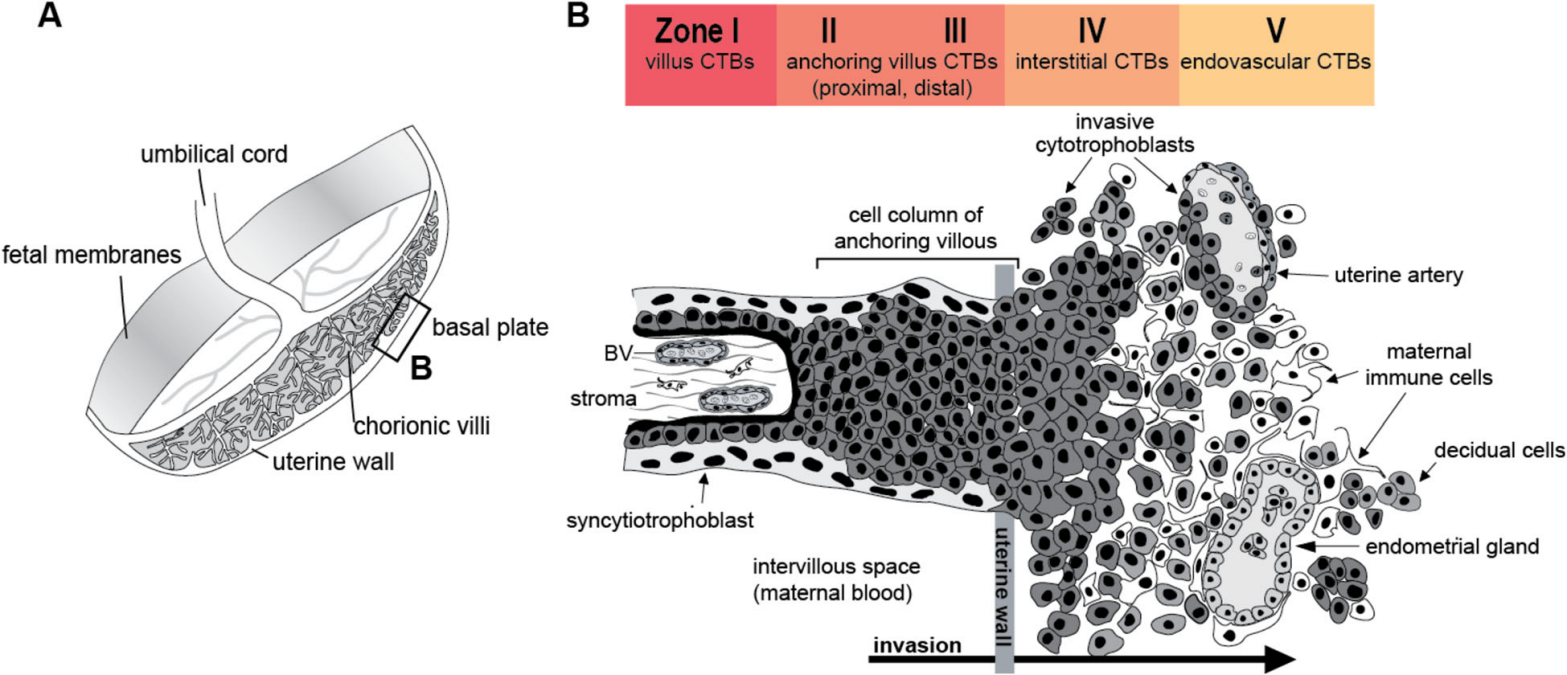
Human placental villous cytotrophoblast (CTB) differentiation at the maternal-fetal interface. **a** Anatomy of the human placenta. Chorionic villi are the functional units. The histology of the boxed area is shown in the panel to the right. **b** Depiction of the maternal-fetal interface at the cellular level. The mononuclear cytotrophoblasts (CTB) of the (early gestation) chorionic villi fuse to become multinuclear syncytiotrophoblasts (STBs), which form the surface of the placenta. Floating villi are perfused by maternal blood. Anchoring villi give rise to invasive interstitial CTBs (CTBi) that emigrate from the chorionic villi via cell columns that attach the placenta to the maternal unit and infiltrate the uterine wall. Maternal cells in this region include the decidua, remodeled uterine blood vessels, which are lined by cytotrophoblasts (CTBe), and immune cells. During vascular invasion, the cells breach both veins and arteries, but they have more extensive interactions with the arterial portion of the uterine vasculature. Here, they replace the endothelial lining and intercalate within the smooth muscle walls of the spiral arteries, producing hybrid vessels that are composed of both embryonic/fetal and maternal cells. Vascular invasion connects the uterine circulation to the intervillous space where maternal blood perfuses the chorionic villi. Immunoreactivity of molecular biomarkers was evaluated in five zones (I-V) corresponding to different stages of CTB differentiation: I) CTB progenitors in floating villi (FV); II) CTBs of the proximal (AVp) and III) distal (AVd) anchoring villi; IV) invading interstitial CTBs (CTBi); and V) endovascular CTBs (CTBe) that remodel the uterine vasculature. Image modified from Maltepe and Fisher, 2015; Damsky et al., 1992 (Damsky et al., 1992; Maltepe and Fisher, 2015)

The placenta plays an essential role in development, supporting embryonic/fetal growth and maternal adaptations to the changing physiology of pregnancy [5, 6]. The human placenta consists of tree-like chorionic villi originating from fetal cells that float in maternal blood (floating villi, FV; Fig. 1). Mononuclear cytotrophoblast (CTB) progenitors (precursor cells) fuse to form multinucleated syncytiotrophoblasts (STBs) that cover the villus surface where they regulate the exchange of nutrients, waste, and gases. A subset of chorionic villi called anchoring villi (AV) attach the placenta to the uterus. CTBs migrate through the AV and form cell columns that are the source of the extravillous (invading the uterus) subpopulation which invades the uterus and maternal vasculature. Vascular CTB remodeling of uterine arteries redirects blood flow to the placenta, maximizing nutrient uptake for embryonic/fetal growth and development over the course of gestation [7]. During normal pregnancy, modulation of numerous molecules characterized by migratory and invasive properties facilitate CTB differentiation along this pathway: extracellular matrix (ECM)-degrading metalloproteinases (e.g., MMPs), cell-ECM (e.g.*,* integrins), and cell-cell adhesion molecules (e.g., cadherins). The net effect is a unique epithelial (non-invasive CTBs of the anchoring villi cell column) to vascular (invasive CTBs that resemble endothelial cells and colonize maternal blood vessels) transformation [8] critical for placental development and function. For example, CTBs upregulate the expression of alpha 5/beta 1 and alpha 1/beta 1 integrin pairs (ITGA5/B1, A1/B1) while down regulating integrin alpha 6/beta 4 (ITGA6/B4) [9]. Furthermore, as CTBs become invasive and enter cell columns, they down regulate CDH1 and up regulate CDH5 (VE-cadherin) [7, 9–11]. Placental development (placentation) is especially critical during mid-gestation, an important time of fetal organogenesis and extreme changes to maternal physiology [4].

Disruption of the balance of adhesion receptor molecules and/or matrix-degrading proteins is associated with pregnancy complications (e.g.*,* preeclampsia, fetal growth restriction, and preterm birth) that can be due to alterations in CTB differentiation which lead to incomplete vascularization [12–16]. For example, in severe preeclampsia, CTB remodeling of maternal vasculature is incomplete, which is thought to contribute to shallow placentation and reduced arterial invasion that are the hallmarks of this syndrome [15]. While specialized subpopulations of immune cells co-occupy the pregnant uterus with placental CTBs, aiding in vascular remodeling [17], excessive white blood cell (leukocyte) infiltration can indicate infection, a major risk factor for preterm birth [18]. Excessive perivillous (fetal side) fibrinoid deposition is also associated with pregnancy complications that either directly or indirectly involve abnormal placentation [19].

Diverse environmental exposures—including heavy metals (i.e., lead and cadmium [20, 21]) and common classes of environmental chemicals (ECs), such as polybrominated diphenyl ethers (PBDEs), per- and polyfluorinated alkyl substances (PFAS), and phthalates—are suspected to contribute to maternal health and pregnancy complications by interfering with placental development and function [22]. However, we know very little about the precise mechanisms by which ECs exert their toxic effects. Despite ongoing efforts to restrict their use as flame retardants, PBDEs remain a global public health concern due to their environmental and biological persistence [23]. Human pregnancy is a period of high sensitivity and susceptibility; meanwhile, PBDEs are commonly identified in placental/fetal tissues [24, 25], and exposures have been associated with maternal health complications [4], adverse birth outcomes [26], and postnatal neurodevelopmental deficits (i.e., lowered IQ [27];). North Americans typically have higher body burden levels due to historically strict flammability standards in the United States (implemented in the 1970s), with some of the highest concentrations ever reported among pregnant women in the State of California [28].

In humans, the relationship between PBDE exposures and pregnancy complications (e.g., preeclampsia) remains undefined. Eslami et al. (2016) found a significant association between total PBDEs and preeclampsia among first mothers in Iran, while no association was detected in a prospective U.S. cohort [29, 30]. In mice and/or human cell lines, PBDEs alter hormone production [31], elicit inflammation [32–34], and promote oxidative stress [32, 35–39]. Data from in vitro studies supports the hypothesis that PBDEs disrupt trophoblast function and placentation. In human primary CTBs (2nd trimester), BDE-47 impairs CTB invasion and migration as well as causes significant changes on the transcriptomic and methylomic level in pathways which regulate placental development [40]. Additional studies using an extravillous trophoblast cell line (HTR-8/SVneo) have shown BDE-47 to be linked with oxidative stress and pro-inflammatory mediators, potential contributors to impaired CTB invasion, as well as placental dysfunction and disease [32, 41]. Together, these studies indicate a potential role for PBDEs in the pathophysiology of adverse pregnancy outcomes via effects on the placenta and highlight functional and molecular perturbations to human placental development that can be evaluated in relation to PBDE exposures.

In this study, we assessed a time period that is a vulnerable window of CTB invasion when remodeling occurs. Perturbations to this developmental process contribute to preeclampsia and other complications usually clinically identified later in pregnancy or at term. Specifically, we examined the relationship between mid-gestational PBDE concentrations and biomarkers of placental development and disease, focusing on molecular and morphological features that: 1) correlate with normal placentation and pregnancy outcomes; 2) are adversely affected in pregnancy complications; and/or 3) are differentially expressed following exposure of primary human CTBs to PBDEs in vitro [40]. The molecules were stage-specific CTB antigens that correlate with differentiation and invasion: integrin alpha 1 (ITGA1), VE-cadherin (CDH5), and metalloproteinase 1 (MMP1). The morphological features evaluated were leukocyte infiltration, fibrinoid deposition, and CTB arterial vascular remodeling. This study will advance new methods and findings that will contribute to better understanding the relationship between biomarkers of chemical exposure and potential biomarkers of placental development and disease (Figure S1).

## Methods

### Study recruitment and sample collection

Non-smoking pregnant women (*n* = 138) with uncomplicated pregnancies undergoing elective terminations during mid-gestation of pregnancy (gestational week [GW] 15–24) were recruited at the Women’s Options Center (WOC) in the San Francisco Bay Area from 2014 to 16. The WOC serves a racially/ethnically diverse low-income population of pregnant women in Northern and Central California who tend to rely on public health insurance coverage for prenatal care. Written and verbal consents were obtained during the clinical visit, followed by administration of a survey questionnaire to each study participant. All study protocols were approved by the UCSF Institutional Review Board prior to the clinical visit.

**Fig. 2:**
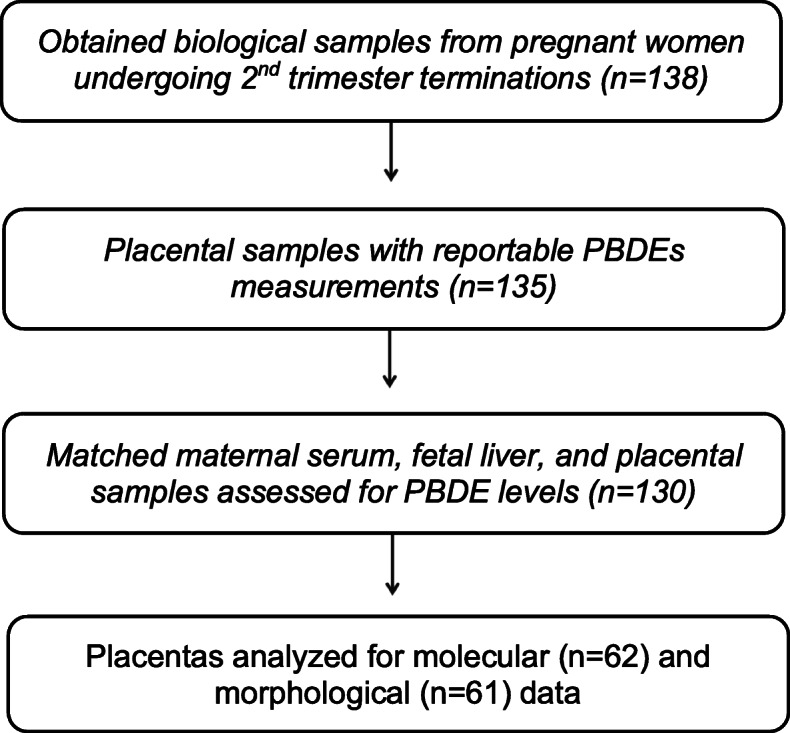
Participant recruitment and biological samples collected at the Women’s Options Center (WOC) in Northern California from 2014 to 16. We obtained tissue samples and questionnaire data from 138 pregnant women undergoing elective terminations during mid-gestation. Polybrominated diphenyl ether (PBDE) levels were measured in 135 placental samples. After excluding twins, PBDE measurements were obtained for 130 matched samples of maternal serum, fetal liver, and the placenta. Then we evaluated a subset of placental samples for molecular (*n* = 62) and morphological (*n* = 61) biomarkers of placental development and disease

We collected the following biological samples—maternal serum, placenta, and fetal liver (*n* = 141, due to three sets of twins)—from study participants during or immediately following the clinical procedure. Fetal sex was determined anatomically and recorded prior to the collection of fetal tissue samples. Chemical PBDE analyses were later performed on *n* = 135 placental samples, with *n* = 130 matched samples (excluding twins) of all three biomatrices (Fig. 2). For the chemical analyses, maternal serum samples were centrifuged at 3000 RPM for 10 min at 4 °C prior to aliquoting and transfer of the serum with glass pipettes into pre-screened (to confirm absence of PBDE) sterilized amber vials (storage at − 80 °C). A subset of placental samples (*n* = 62) was further processed and evaluated for three molecular and morphological features (see below). The placental subset was selected based on the highest and lowest PBDE exposure levels to maximize our ability to detect differences, which we expected would reduce selection bias and enhance generalizability across the whole population. Population characteristics between the subset assessed for placental biomarkers (*n* = 62) and the whole population (*n* = 130) were similar. Placental biopsies contained a section of the basal plate (which represents the maternal-fetal interface) and includes placental regions covered in our analysis (chorionic villi and decidua).

Placental tissues were submerged in medium (DME/H-21 [Gibco], 12.5% fetal bovine serum [Hyclone], 1% glutamine plus [Atlanta Biologicals], 1% penicillin/streptomycin [Invitrogen], and 0.1% gentamicin [Gibco]); dissected into 1 x cm^3^ pieces; fixed with 3% paraformaldehyde (PFA); and frozen in Optimal Cutting Temperature (OCT) medium (Sakura Finetek, SA62550–01) in a cryomold at -80 °C [42]. Placental biopsies were later sectioned (5 μM thickness) using a cryostat (Leica) and placed on glass slides for molecular and morphological assessments.

### Chemical exposure assessment

Nineteen PBDE congeners (including BDE-17, − 28, − 47, − 66, − 85, − 99, − 100, − 153, − 154, − 183, − 196, − 197, − 201, − 202, − 203, − 206, − 207, − 208, and − 209) were analyzed in maternal serum, placenta, and fetal liver by the Environmental Chemistry Laboratory at California’s Department of Toxic Substances Control in Berkeley, CA, using gas chromatography–high-resolution mass spectrometry (GC–HRMS, DFS, ThermoFisher, Bremen, Germany). Isotopically-labeled internal surrogate mix standards (IS) were used for quantitation (Wellington Laboratories, Inc., Guelph, Ontario, Canada).

Thawed serum samples (1 mL) were spiked with carbon-labeled internal standards (^13^C_12_-BDE-28, 47, 99, 153, 154, 183, 197, 207, and 209), with 4 mL each of formic acid and water added. Serum samples were vortexed and loaded into an automated sample extraction system (RapidTrace, Biotage; Uppsala Sweden). Oasis HLB cartridges (3 cc, 500 mg, Waters Corp.; Milford, MA) were used for sample extraction and acidified silica (500 °C prebaked, manually packed, 3 cc) for sample extraction cleanup. Final eluates were concentrated 10-fold using an automated nitrogen evaporation system (TurboVap LV, Biotage; Uppsala, Sweden) and spiked with a ^13^C_12_-PCB-209 recovery standard [43, 44]. Total cholesterol and triglycerides were measured enzymatically by Boston Children’s Hospital (Boston, MA) and subsequently used to calculate the total serum lipid concentration for each study participant using the Phillips formula [45]. Fetal liver and placental samples were analyzed using our liver analytical method with slight modification [25]. Before sample extraction, only placenta samples were lyophilized. Briefly, samples were homogenized and spiked with the same internal standards (listed above). Samples were then denatured with hydrochloric acid and extracted with 1:1 hexane:methyl tert-butyl ether (MTBE). Aqueous potassium chloride solution was added to each sample extract to remove potentially co-extracted aqueous compounds and the organic layer was re-extracted and dried in a pre-weighed, pre-baked aluminum weighing dish for lipid content determination via gravimetric analysis. Samples were then reconstituted in hexane and lipids were removed using concentrated sulfuric acid, followed by cleanup with acidified silica (500 °C prebaked, manually packed, 3 cc) on automated SPE system (RapidTrace, Biotage; Uppsala Sweden).

### Assessment of stage-specific antigens and morphological endpoints

Molecular and morphological placental features were evaluated independently by two investigators who were blinded to PBDE exposure levels. We assessed molecular immunoreactivity of CTBs at the maternal-fetal interface with antibodies specific for integrin alpha 1 (ITGA1), VE-cadherin (CDH5) or metalloproteinase 1 (MMP1) in five stages of trophoblast differentiation: Zone I) CTBs resident in FV; Zone II) CTBs in the proximal (p) regions of AV cell columns; Zone III) CTBs within the distal (d) regions of AV cell columns; Zone IV) interstitial invasive/extravillous CTBs (iCTB); and Zone V) endovascular CTBs (eCTB) (Fig. 1). We selected ITGA1 and CDH5 based on previous in vitro and in vivo literature demonstrating the importance of these adhesion receptor molecules in placental development and function (specifically regarding invasive and endovascular CTB differentiation pathways that are critical for vascular remodeling) as well as their potential disease associations. MMP1 is a metalloproteinase matrix-degrading enzyme that facilitates CTB migration/invasion during placentation [7, 9, 46] (Table S1 and Figures S3, S4, S5). We immunolocalized ITGA1, CDH5, and MMP1 in each placental zone using published methods [47]. The antibodies and dilutions that were used for this purpose are described in (Table S2). CTBs were identified by reactivity with CK7. Batch effects which influence immunofluorescence (IF) intensity were continuously monitored by including serial sections from the same two placental samples in each assessment. Slides were imaged using an upright Leica DFC450 microscope equipped with a camera and Leica Advanced Fluorescence Application Suite Ver. 3.2 (Leica Microsystems). Immunoreactivity was scored using a previously established semi-quantitative approach in which two independent reviewers categorized immunofluorescence into three broad pre-defined categories (rather than continuous measures) based on the percentage of CTBs that reacted with each antibody: 1) <  25% (−); 2) 25–75% (−/+); and 3) > 75% (+) [47].

Placental biopsies were also stained with Hematoxylin and Eosin (H&E) to evaluate morphological features: (1) average total number of white blood cells (WBC) present in basal plate (BP) per 10X field; (2) percent of FV with perivillous fibrinoid deposits (~ 60 villi evaluated per field); 3) fibrinoid deposition at the utero-placental junction; and (4) total number of modulated uterine spiral arteries which contained CTBs (defined as presence of > 50% endovascular CTBs in the lining of uterine spiral arteries) (Table S1 and Figure S5). All values were based on examination of the entire placental section at 10X resolution (~ 6–10 independent fields). In total, we evaluated 62 placentas; however, the sample size for each molecular and morphological endpoint varied depending on whether tissue sections captured the key structures of interest, most notably uterine arteries. For example, eCTB were confined to placental tissue sections that contained uterine arteries (*n* = 29–42); pAV and dAV CTBs were observed in 51–53 samples, and iCTB were found in 60 samples (Table S3). For morphological assessments, one sample was lost while processing (*n* = 61 placentas evaluated). Images were acquired using a bright field Leica DFC450 microscope equipped with a camera (Figure S5).

### Statistical analysis

We calculated detection frequencies for all 19 PBDE congeners in matched samples of maternal serum, fetal liver, and placental tissues obtained in this study (2014–16; *n* = 130) (Table S4). We normalized wet-weight PBDE concentrations to total lipid levels in order to account for possible measurement error related to estimating PBDE exposure from biomonitoring data, since biological proxies of exposure to PBDEs and other lipophilic compounds can vary systematically with population characteristics (e.g., lipid content) rather than the PBDE exposures they represent [48, 49]. For five PBDE congeners (BDE-28, − 47, − 99, − 100, and − 153) detected in > 50% of placental samples (*n* = 135), we further calculated wet-weight and lipid-adjusted descriptive statistics, including geometric mean, interquartile range (25th–75th percentile), and range (min–max). We also computed a summary PBDE metric by adding four of congeners (ΣPBDE4 = BDE-47, − 99, − 100, and − 153) with detection frequencies > 50% in all maternal-fetal tissues, in order to facilitate results comparison with maternal serum and fetal liver in secondary analyses. We used maximum likelihood estimation (MLE) assuming a log-normal distribution to account for PBDE concentrations below the laboratory method detection limit (MDL) [50]. Statistical analyses were performed using R (Version 3.5.1) [51], with significance defined as *p* < 0.05 and marginal significance as *p* < 0.10 (two-sided tests).

We examined how PBDE concentrations varied across categorical population characteristics, including maternal age (< 20, 20–24, 25–29, ≥ 30 years), gestational age (< 19, 19–21, ≥ 21 weeks), body mass index (BMI; < 25, 25–30, ≥ 30 kg/m2), parity (0 or ≥ 1 live births), fetal sex (male or female), education (≤ High School or ≥ Some college), type of insurance (public or private/self-pay), race/ethnicity (Latina/Hispanic, Non-Hispanic Black, Non-Hispanic White, Asian/Pacific Islander), birth country (U.S. or foreign born), and sample collection year (2014, 2015, 2016). Although sample size precluded multivariable analysis, we examined population characteristics in separate models with our exposure (PBDE concentrations) and outcome of interest (placental biomarkers). For example, although fetal sex could conceivably be an effect modifier of PBDE effects on placental development and function, we did not observe variation with exposures or outcomes in this study.

In addition, we computed descriptive statistics for molecular and morphological placental biomarker data. We natural log-transformed average WBC counts prior to statistical analysis based on visual inspection of right-skewed distribution as well as results from the Shapiro-Wilk statistical test. One observation had a WBC count of zero which we set to 0.01 prior to log transformation. We calculated the percent of modulated uterine spiral arteries by dividing the number of uterine spiral arteries with > 50% endovascular CTB modulation by the total number of uterine spiral arteries observed. If the total number of uterine spiral arteries (i.e., the denominator) was zero or less than two, the denominator and percent modulation were re-coded as missing. Then we divided % CTB-modulated uterine blood vessels (mBV) at the median and categorized the variable into two groups (low and high). We modeled molecular immunoreactivity categorically rather than continuously based on the three pre-defined categories that were scored by two independent reviewers in the laboratory (as described in section 2.3): (1) <  25% (−); 2) 25–75% (−/+); and 3) > 75% (+).

Kendall’s Tau Correlation Coefficient [52], a non-parametric measure of correlation that compares concordant and discordant pairs of ordered data to determine the extent of increasing or decreasing covariation between two variables, was used to evaluate monotonic relationships: 1) among molecular and morphological placental biomarkers; and 2) between placental biomarkers (molecular and morphological) and placental PBDE concentrations (wet-weight and lipid-normalized). We calculated the correlation between placental biomarkers and PBDE concentrations using the censored version of Kendall’s tau [53]. We adjusted for multiple comparisons by estimating the false discovery rate (FDR) using the Benjamini and Hochberg method [54].

To compare PBDE levels across immunoreactivity groups, we tested group mean differences (assuming a log-normal PBDE distribution) using MLE to account for left-censored PBDE data [50]. From these bivariate censored regression/ANOVA models, we calculated the pairwise percent (%) differences in PBDE concentrations as (e^*β*^ − 1) ∗ 100 and the 95% confidence interval (95% CI) as (e^*β* ± 1.96 × SE^ − 1) ∗ 100, where the referent group comprised placental samples in the < 25% (−) immunoreactivity group. The beta coefficient from these models represents the difference in PBDE levels on the lognormal scale between moderate or high immunoreactivity groups and the referent group, while e^*β*^ represents the ratio of average PBDE concentrations between immunoreactivity groups. We also used the Fisher’s exact test to examine whether molecular immunoreactivity varied by high/low PBDE exposure (where PBDE levels were divided at the median and categorized into high vs. low exposure groups).

In addition to assessing correlations between placental PBDE concentrations and placental biomarkers, we also re-examined placental biomarker associations with fetal liver and maternal serum PBDE concentrations, since we previously found moderate correlation between congeners across maternal-fetal tissues during mid- gestation, with cross-tissue correlations ranging from 0.18 (BDE-99) to ≥0.50 (BDE-47, − 100, and − 153) (*p* < 0.0001) [55].

## Results

The majority of women in our study population (*n* = 135) were < 30 yrs. old (79%); participants 20–24 yrs. old were the largest age subgroup (43%; Table 1). Gestational age ranged from ~ 15–24 wks (median = 20 wks). Most women were classified as overweight (28%; BMI = 25–30 kg/m^2^) or obese (29%; BMI ≥ 30 mg/m^2^). Approximately 60% of participants had at least one prior live birth and 54% of the pregnancies were with a male fetus. Education level was evenly divided between high school attendance or graduation and some post-secondary schooling. Most women used public rather than private health insurance. The study population was racially/ethnically diverse (> 50% Latina/Hispanic and non-Hispanic Black) (Table 1).

**Table 1:** Population characteristics and placental PBDE levels during mid-gestation at the Women’s Options Center (WOC) in Northern California, 2014–16 (*n* = 135)

		***Wet-weight BDE-47***		***Lipid-adjusted BDE-47***		***Wet-weight ∑ PBDE4***^***a***^		***Lipid-adjusted ∑ PBDE4***^***a***^	
Population Characteristic	*n (%)*	*GM (95% CI)*	*p-Value*^*b*^	*GM (95% CI)*	*p-Value*^*b*^	*GM (95% CI)*	*p-Value*^*b*^	*GM (95% CI)*	*p-Value*^*b*^
**Maternal Age**									
< 20 years	19 (14)	0.10 (0.08, 0.14)	0.910	11.1 (8.4, 14.6)	0.945	0.20 (0.16, 0.26)	0.922	21.6 (16.9, 27.5)	0.893
20–24 years	58 (43)	0.10 (0.08, 0.14)		10.8 (7.8, 14.9)		0.20 (0.15, 0.27)		20.9 (15.7, 27.8)	
25–29 years	30 (22)	0.10 (0.07, 0.14)		11.1 (7.7, 15.8)		0.20 (0.14, 0.27)		22.1 (16.1, 30.4)	
≥ 30 years	29 (21)	0.10 (0.07, 0.15)		11.3 (7.8, 16.2)		0.19 (0.14, 0.26)		21.0 (15.3, 29.0)	
**Gestational Age**									
< 19 weeks	37 (27)	0.11 (0.09, 0.13)	0.255	11.6 (9.5, 14.1)	0.601	0.21 (0.18, 0.25)	0.440	22.4 (18.8, 26.8)	0.756
19–21 weeks	51 (38)	0.11 (0.09, 0.14)		11.3 (8.7, 14.8)		0.21 (0.16, 0.26)		21.3 (16.8, 26.9)	
≥ 21 weeks	48 (35)	0.09 (0.07, 0.12)		10.2 (7.8, 13.4)		0.18 (0.15, 0.23)		20.5 (16.2, 26.0)	
**Body Mass Index (BMI)**									
Normal/Under (< 25 kg/m^2^)	59 (43)	0.10 (0.08, 0.11)	0.602	10.8 (9.2, 12.7)	0.795	0.19 (0.16, 0.22)	0.594	21.1 (18.3, 24.3)	0.652
Overweight (25–30 kg/m^2^)	38 (28)	0.11 (0.09, 0.14)		10.6 (8.1, 13.7)		0.21 (0.17, 0.26)		20.2 (16.1, 25.5)	
Obese (≥ 30 kg/m^2^)	39 (29)	0.10 (0.08, 0.13)		11.6 (9.0, 15.0)		0.20 (0.16, 0.25)		22.7 (18.2, 28.4)	
**Parity**									
0 live births	55 (40)	0.09 (0.08, 0.11)	0.309	9.9 (8.4, 11.6)	0.240	0.19 (0.16, 0.21)	0.443	19.6 (16.9, 22.6)	0.325
≥ 1 live births	81 (60)	0.11 (0.09, 0.13)		11.9 (9.6, 14.7)		0.21 (0.17, 0.25)		22.6 (18.7, 27.4)	
**Fetal Sex**									
Male	73 (54)	0.10 (0.09, 0.12)	0.955	10.2 (8.8, 11.8)	0.379	0.20 (0.18, 0.23)	0.942	20.4 (18.0, 23.3)	0.659
Female	63 (46)	0.10 (0.09, 0.13)		11.9 (9.6, 14.7)		0.20 (0.16, 0.23)		22.3 (18.5, 26.9)	
**Education**									
≤ High School	67 (49)	0.11 (0.10, 0.13)	0.260	11.9 (10.2, 13.8)	0.415	0.22 (0.19, 0.25)	0.177	23.1 (20.2, 26.3)	0.308
≥ Some college	68 (50)	0.09 (0.08, 0.12)		10.3 (8.3, 12.7)		0.18 (0.15, 0.22)		19.9 (16.5, 24.0)	
**Insurance**									
Public	98 (72)	0.11 (0.10, 0.12)	0.364	11.7 (10.3, 13.3)	0.159	0.21 (0.19, 0.23)	0.222	22.7 (20.4, 25.3)	**0.066**
Private/Self-pay	37 (27)	0.09 (0.07, 0.11)		9.3 (7.3, 11.7)		0.17 (0.14, 0.21)		17.7 (14.4, 21.8)	
**Race/ethnicity**									
Latina/Hispanic	45 (33)	0.10 (0.09, 0.12)	0.102	10.2 (8.6, 12.1)	**0.038**	0.19 (0.16, 0.22)	**0.011**	18.8 (16.2, 21.8)	**0.001**
Non-Hispanic Black	31 (23)	0.12 (0.09, 0.16)		13.7 (10.4, 17.9)		0.24 (0.19, 0.31)		27.6 (21.9, 34.8)	
Non-Hispanic White	20 (15)	0.09 (0.07, 0.13)		10.6 (7.8, 14.4)		0.18 (0.14, 0.23)		20.2 (15.4, 26.3)	
Asian/Pacific Islander	17 (12)	0.09 (0.06, 0.12)		9.2 (6.6, 12.7)		0.16 (0.12, 0.21)		17.0 (12.8, 22.6)	
**Birth Country**									
U.S. born	71 (52)	0.11 (0.10, 0.13)	0.114	11.8 (10.3, 13.6)	0.222	0.21 (0.19, 0.23)	**0.009**	22.4 (19.9, 25.3)	**0.025**
Foreign born	9 (7)	0.07 (0.05, 0.11)		8.3 (5.5, 12.4)		0.12 (0.08, 0.17)		13.5 (9.4, 19.3)	
**Collection Year**									
2014	33 (24)	0.11 (0.09, 0.13)	**0.032**	13.8 (11.3, 16.9)	**0.004**	0.20 (0.17, 0.24)	0.125	24.8 (20.7, 29.7)	**0.037**
2015	93 (68)	0.10 (0.08, 0.12)		9.8 (7.7, 12.3)		0.19 (0.16, 0.24)		19.6 (15.9, 24.3)	
2016	10 (7)	0.16 (0.10, 0.26)		15.9 (10.0, 25.3)		0.29 (0.19, 0.43)		28.0 (18.4, 42.5)	
*Total (pooled)*^*b*^	*135 (100)*	*0.10 (0.09; 0.03–0.92)*		*11.0 (9.7; 3.1–107)*		*0.20 (0.15; 0.07–1.3)*		*21.3 (16.4; 5.8–152)*	

*GM* Geometric mean, *IQR* Interquartile range (25th–75th percentile)

† Number (%) missing = 56 (41), 1 (0.7), 1 (0.7), and 23 (17) for birth country, education, insurance, and race/ethnicity, respectively

^*a*^*∑ PBDE4 = BDE-47 + BDE-99 + BDE-100 + BDE-153*

^*b*^*P*-values from ANOVA test of mean difference in Ln PBDE levels between population characteristic subgroups. *P* < 0.10 bolded

^*c*^Summary statistics of placental PBDE levels in total (pooled) study population, including: GM (IQR; Min–Max)

PBDEs were detected in all placental samples, with the range of wet-weight PBDE concentrations spanning more than one order of magnitude, and lipid-adjusted PBDE levels greater than wet-weight concentrations by almost two orders of magnitude (Table 1). Placental PBDE concentrations did not vary by maternal age, gestational age, BMI, parity, fetal sex, education, or insurance type (*p* > 0.05). In contrast, we observed placental PBDE exposure differences based on race/ethnicity, birth country, and sample collection year. More specifically, placental PBDE concentrations were highest among US-born participants, Non-Hispanic Black women, and in earlier collection years (2016 > 2014 > 2015) (Table 1), which is consistent with previous findings although birth country differences should be interpreted in the context of missing data for most study participants [55].

**Fig. 3:**
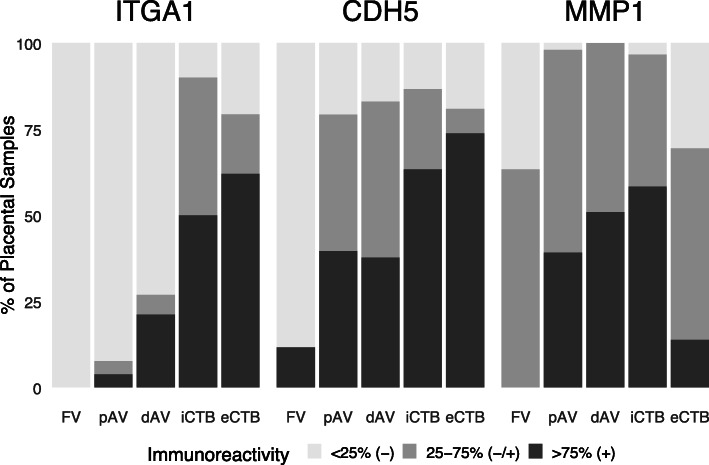
Immunoreactivity of ITGA1, CDH5, and MMP1 as a function of cytotrophoblast (CTB) differentiation/invasion during mid-gestation (*n* = 62). The following cell types were scored: 1) CTBs in floating villi (FV); anchoring villi CTBs in the 2) proximal (pAV) and 3) distal (dAV) regions of cell columns; 4) invasive interstitial CTBs (iCTB); and 5) endovascular CTBs (eCTB). CTB immunoreactivity in each region was scored based on the percent of cells that stained for each biomarker in that region: 1) < 25% (−), 2) 25–75% (−/+), or 3) > 75% (+). Staining patterns varied by antigen and CTB subtype (*p* < 0.001; Fisher’s exact test)

ITGA1, CDH5, and MMP1 immunoreactivity was in accord with previously published results [7, 9, 46]. These stage-specific antigens were upregulated as CK-positive CTBs exited the placenta and invaded the uterine wall (*p* < 0.001) (Fig. 3 and Table S3). Staining patterns varied by region and antibody target. For example, ITGA1 immunoreactivity was minimally detected in FV CK+ CTBs and upregulated as the cells differentiated and invaded, increasingly detected in association with pAV, dAV, iCTB, and eCTB CTBs. CDH5 immunoreactivity had a similar pattern except that FV CK+ CTBs in a few samples expressed this cell-cell adhesion molecule. With the exception of eCTB, MMP1 was more widely expressed by the CTB subpopulations on which we focused.

On average, WBC infiltration was 212 cells ±174 (Table 2). The percentage of FV with associated fibrin deposits was 13.4 ± 10.4%, while fibrinoid deposition in the BP delimited 22.6 ± 19.6% of the CTB-decidual boundary. In a subset of placental biopsies (*n* = 38) with at least three uterine spiral arteries, 71.7 ± 30.4% of blood vessels were modulated, defined as having > 50% CK-positive CTBs within the walls or lumens. As expected, given the narrow range in gestational age, correlations between morphological features and weeks of pregnancy were not significant. The exception was possible evidence of an inverse relationship between perivillous fibrinoid deposition and gestational age (*τ* = − 0.28; *p* = 0.001) (Table 2).

**Table 2:** Descriptive statistics of morphological placental biomarkers and correlation^*a*^ with gestational age during mid-gestation (*n* = 61)

							*Gestational Age (Weeks)*	
***Morphological Biomarker***	***n***	***Mean***	***Median***	***SD***	***Min***	***Max***	***Tau***	***p-Value***
Average White Blood Cell / image	61	212	157	174	0.00	950	−0.12	0.179
% Floating Villi with Fibrinoid Deposition (binary)	61	13.4	11.8	10.4	0.10	47.1	−0.28	0.001
% Fibrinoid Deposition in Basal Plate (area)	61	22.6	20.0	19,6	0.00	78.0	0.02	0.866
% CTB-modulated Uterine Spiral Arteries	38	71.7	80.6	30.4	0.00	100	−0.14	0.240

*CTB* Cytotrophoblast, *SD* Standard deviation

^*a*^ Non-parametric rank order correlation assessed using Kendall’s Tau Correlation Coefficient

The rank order correlation of ITGA1, CDH5, and MMP1 immunoreactivity varied among molecules and placental region/cell type (Tables S5 and S6). Molecular markers were more highly correlated between CTB subtypes that were closely related on a developmental continuum and located in proximity to one another (iCTB and eCTB; pAV and dAV). For example, previous studies showed that the stage-specific antigens of interest are upregulated as the cells leave the placenta and invade the uterine wall [7, 9, 46]. Accordingly, MMP1 immunoreactivity in CK+ cells was positively correlated between CTBs in pAV and dAV (*τ* = 0.42), dAV CTBs and iCTB (*τ* = 0.46), and iCTB and eCTB (*τ* = 0.32); there was no correlation between CTBs in FV and pAV. As expected, we found ITGA1 and CDH5 were similarly regulated. Interestingly, while we found no-to-low correlation between ITGA1 and CDH5 immunoreactivity, regardless of placental region/cell type, MMP1 immunoreactivity was modestly correlated with the other two molecular markers. We found little association among the morphological endpoints except for a positive correlation between fibrinoid deposition in the FV and basal plate (*τ* = 0.24, *p* = 0.01). In the absence of additional data, we were unable to determine whether strong correlations exist between molecular and morphological biomarkers, although this analysis was limited by sample size. However, several trends were notable, including a consistent inverse correlation between immunoreactivity for MMP1 (*τ* = − 0.27), ITGA1 (*τ* = − 0.21), and CDH5 (*τ* = − 0.26) in dAV and WBC count. There was also an inverse association between expression of these stage specific antigens and fibrinoid deposition in some regions. For example, reduced MMP1 immunoreactivity (*τ* = − 0.29) in CTBs of the dAV and the percent of floating villi with fibrinoid deposits and vice versa. Adjusting for multiple comparisons did not substantially change overall correlation patterns between molecular and morphological biomarkers (Tables S5 and S6).

In general, we did not observe significant correlations between PBDE concentrations (BDE-47 or ΣPBDEs) and most molecular or morphological biomarkers (Table S7). The exception was an inverse relationship between ITGA1 immunoreactivity in eCTB with levels of most PBDE congeners, individually and as a group. For example, BDE-47 was inversely correlated with eCTB ITGA1 (*τ* = − 0.25). Similar inverse relationships were observed for congeners − 99, − 100, and ΣPBDE4 but were attenuated for BDE-28 and BDE-153. We also found a positive correlation between BDE-28 and MMP1 immunoreactivity in iCTB (*τ* = 0.18). Correlations with lipid-adjusted PBDE concentrations were similar and thus not reported. Adjusting for multiple comparisons revealed the potential for false positive findings (Table S7), suggesting these observations could be strengthened by analyzing more samples.

**Fig. 4:**
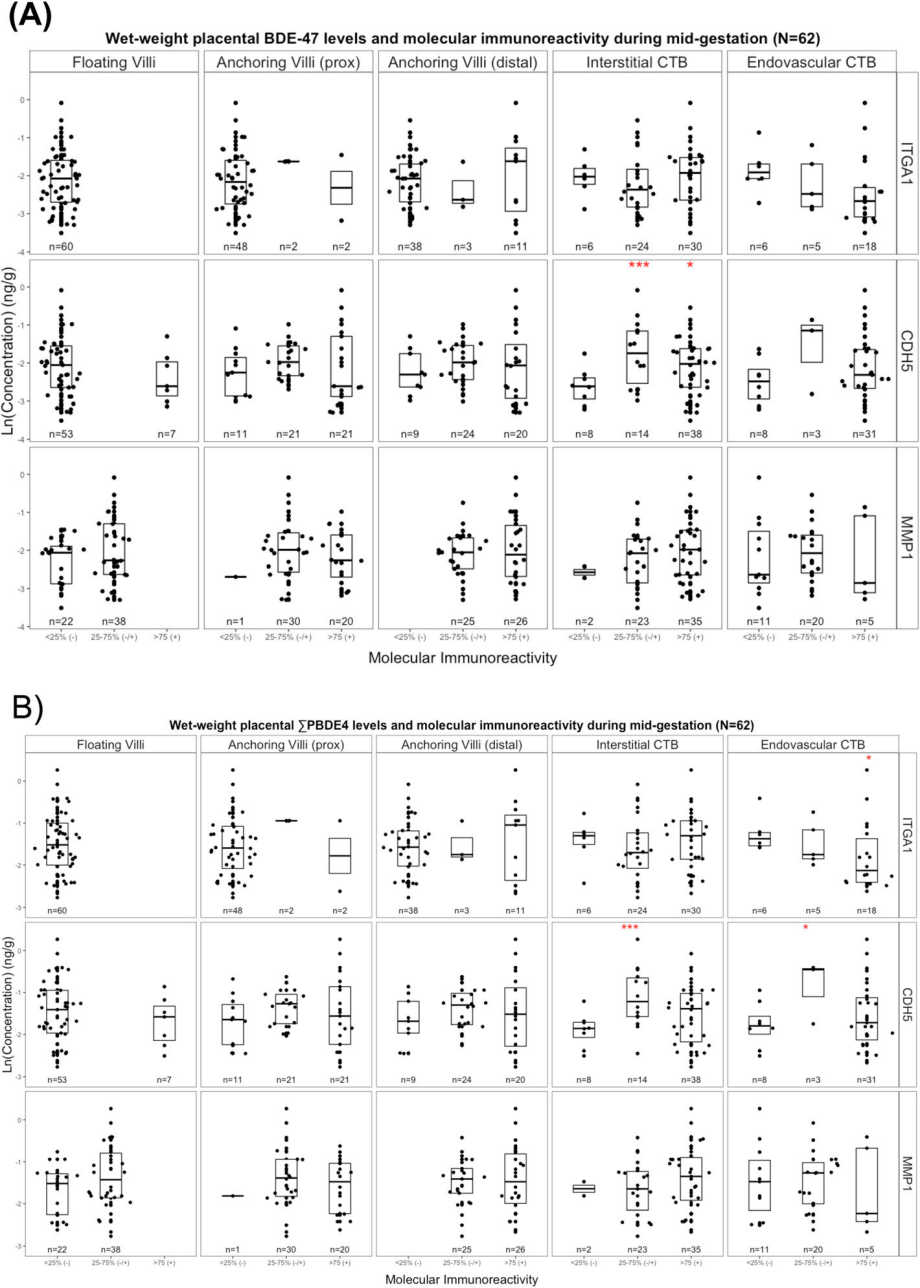
Wet-weight placental (**a**) BDE-47 and (**b**) åPBDE4 molecular immunoreactivity by placental region/cell type during mid-gestation (*n* = 62). åPBDE4 = BDE-47 + − 99 + − 100 + − 153. Red asterisks denote significance or marginal significance (where: *p < 0.10; **p < 0.05; and ***p < 0.01) from bivariate censored MLE regression models evaluating the percent (%) difference in placental PBDE levels between moderate or high immunoreactivity compared to low immunoreactivity/referent group: 25–75% (−/+) or > 75% (+) compared to < 25% (−)

The analysis revealed a similar inverse trend in the magnitude of ITGA1 immunoreactivity and placental concentrations of PBDE congeners. For example, placentas with the highest levels of ITGA expression in eCTB had 42% (95% CI: − 69–7.2%) lower levels of ∑PBDE4 compared to the referent group (Table 3 and Fig. 4). The magnitude and direction of association with CTBe ITGA-1 immunoreactivity was consistent for all congeners, despite ANOVA tests which did not reveal an overall difference between groups (*p* > 0.05). We also found a positive association between wet-weight and lipid-adjusted placental BDE-47 or ΣPBDE4 concentrations and CDH5 immunoreactivity in iCTB (*p* < 0.05). The same relationship held for eCTB, although at a lower level of statistical significance (Table 3 and Fig. 4).

**Table 3:** Percent (%) difference in placental PBDE levels between molecular immunoreactivity groups for each biomarker within each placental region/cell type during mid-gestation (*n* = 62)

		Wet-weight BDE-47 (ng/g)		Wet-weight ∑PBDE4 (ng/g)		Lipid-adjusted BDE-47 (ng/g lipid)		Lipid-adjusted ∑PBDE4 (ng/g lipid)	
***Immunoreactivity***	*n*	*% diff (95% CI)*^*a*^	*p-value*^*b*^	*% diff (95% CI)*^*a*^	*p-value*^*b*^	*% diff (95% CI)*^*a*^	*p-value*^*b*^	*% diff (95% CI)*^*a*^	*p-value*^*b*^
**Anchoring Villi (proximal)**									
*ITGA1* < 25% (−)	48	Referent	0.616	Referent	0.433	Referent	0.813	Referent	0.724
25–75% (−/+)	2	68 (−44, 403)		82 (−32, 385)		38 (−56, 331)		49 (− 46, 316)	
> 75% (+)	2	−16.(−72, 151)		−21 (−70, 110)		23 (−61, 283)		15 (−59, 221)	
*CHD5* < 25% (−)	11	Referent	0.502	Referent	0.404	Referent	0.441	Referent	0.357
25–75% (−/+)	21	35 (−20, 130)		39 (−14, 125)		39 (−20, 142)		43 (−13, 136)	
> 75% (+)	21	13 (−34, 91)		21 (−25, 94.8)		11 (−36, 93)		19 (−28, 96)	
*MMP1* < 25% (−)	1	Referent	0.395	Referent	0.472	Referent	0.741	Referent	0.862
25–75% (−/+)	30	109 (−51, 797)		56 (−58, 481)		69 (−64, 700)		26 (−69, 413)	
> 75% (+)	20	67 (−61, 626)		2 (−6, 375)		51 (−69, 627)		14 (−72, 371)	
**Anchoring Villi (distal)**									
*ITGA1* < 25% (−)	38	Referent	0.759	Referent	0.837	Referent	0.599	Referent	0.739
25–75% (−/+)	3	−19 (−68, 102)		4.1 (−54, 138)		−28 (−72, 83)		− 7.8 (− 61, 116)	
> 75% (+)	11	15 (−32, 94)		15 (−28, 85)		19 (−30, 104)		20 (−26, 95)	
*CHD5* < 25% (−)	9	Referent	0.670	Referent	0.550	Referent	0.662	Referent	0.542
25–75% (−/+)	24	29 (−26, 125)		32 (−20, 120)		31 (−27, 135)		35 (−21, 128)	
> 75% (+)	20	17 (−34, 108)		19 (−29, 100)		19 (−35, 117)		21 (−30, 108)	
*MMP1* < 25% (−)	0	Referent	0.901	Referent	0.944	Referent	0.661	Referent	0.697
25–75% (−/+)	25	−9.1 (−40, 37)		−6.2 (− 35, 36)		−18 (−47, 26)		−15 (−43, 24)	
> 75% (+)	26	–		–		0.0 (0.0, 0.0)		0.0 (0.0, 0.0)	
**Interstitial CTB**									
*ITGA1* < 25% (−)	6	Referent	0.622	Referent	0.556	Referent	0.881	Referent	0.863
25–75% (−/+)	24	−16 (−58, 66)		−17.1 (−55, 53)		−3.5 (−53, 98)		−4.4 (−49, 81)	
> 75% (+)	30	2.3 (−48, 101)		1.07 (−45, 85)		7.6 (−47, 117)		6.3 (−43, 99)	
*CHD5* < 25% (−)	8	Referent	0.032**	Referent	0.028**	Referent	0.018**	Referent	0.019**
25–75% (−/+)	14	138 (26, 350)***		115 (22, 279)***		163 (37, 405)***		137 (33, 325)***	
> 75% (+)	38	63 (−6.6, 185)*		42 (−14, 133)		102 (14, 257)**		75 (5.3, 192)**	
*MMP1* < 25% (−)	2	Referent	0.372	Referent	0.350	Referent	0.693	Referent	0.727
25–75% (−/+)	23	41 (−53, 324)		−0.38 (−63, 167)		36 (−57, 330)		−4.1 (− 66, 168)	
> 75% (+)	35	77 (−40, 425)		29 (−51, 242)		53 (−51, 376)		12 (−59, 208)	
**Endovascular CTB**									
*ITGA1* < 25% (−)	6	Referent	0.303	Referent	0.211	Referent	0.518	Referent	0.386
25–75% (−/+)	5	−30 (−73, 80)		−22 (−65, 73)		−9.3 (− 66, 145)		1.0 (−57, 135)	
> 75% (+)	18	−44 (−73, 16)		− 42 (−69, 7.2)*		−34 (− 69, 44)		− 31 (− 64, 32)	
*CHD5* < 25% (−)	8	Referent	0.256	Referent	0.175	Referent	0.145	Referent	0.151
25–75% (−/+)	3	140 (−16, 590)		146 (−2.8, 521)*		134 (−21, 588)		139 (−8.3, 522)*	
> 75% (+)	31	42 (−24, 162)		23 (−29, 112)		82.4 (−3.2, 243)		59 (−9.5, 178)	
*MMP1* < 25% (−)	11	Referent	0.883	Referent	0.846	Referent	0.847	Referent	0.736
25–75% (−/+)	20	12 (−40, 110)		1.1 (−42, 77)		8.0 (− 43, 106)		−2.8 (−45, 73)	
> 75% (+)	5	−6.6 (−6, 130)		−19 (−64, 83)		−16 (−67, 113)		−27 (− 68, 67)	

*p < 0.10. ** *p* < 0.05. *** *p* < 0.01. Significant and marginally significant results bolded

^*a*^Calculated from bivariate censored regression models, where referent group included placental samples with trophoblast cells (identified as cytokeratin+ cells) that did not stain (−) or stained only minimally (0–25%) for antigen specific antibodies corresponding to each molecular biomarker

^*b*^*P*-value from ANOVA test of mean difference in Ln PBDE level across three immunoreactivity groups

We observed a similar pattern of association with PBDEs in maternal serum and the fetal liver, with ITGA1 (eCTB) and CDH5 (iCTB) correlations that were strongest in the fetal liver, and minimal evidence of correlation with morphological biomarkers (Supplemental Spreadsheet).

## Discussion

This is the largest study to our knowledge which characterizes a combination of molecular and morphological biomarkers measured with high resolution and precision directly in placental tissues during the second trimester. We additionally examined their relationship to placental (and maternal-fetal) PBDE concentrations for a first ever in vivo investigation of PBDEs and mechanistic effects on placentation during a vulnerable window of development. We immunolocalized for three molecules–integrin alpha 1 (ITGA1), VE-cadherin (CDH5), and metalloproteinase 1 (MMP1)–in CTBs within six regions of the basal plate and confirmed their increased presence as they invaded into the maternal unit. We also found possible inverse and positive PBDE associations with ITGA1 immunoreactivity (endovascular CTBs) and CDH5 immunoreactivity (interstitial and endovascular CTBs), respectively, which are molecular hallmarks of placental development and function during pregnancy.

ITGA1 and CDH5 play key roles in CTB differentiation and have potential disease associations (e.g., preeclampsia) [7, 9, 11]. We also selected to examine MMP1 in vivo since our previous in vitro analysis revealed upregulated expression of *MMP1* in differentiating CTBs exposed to BDE-47 [40]. In agreement with previously published data [42], ITGA1 was upregulated in cell columns and primarily expressed in extravillous CTBs, especially in the interstitial and endovascular regions of the basal plate (iCTB and eCTB). Corresponding with previous observations [7], CDH5 had a similar expression pattern. MMP1 is thought to be involved in extracellular matrix (ECM) breakdown during CTB invasion [56]. We confirmed upregulated anti-MMP1 reactivity associated with CTBs of the cell column, iCTB, and eCTB, Thus, the stage-specific antigens we profiled are upregulated as part of the molecular program that enables placental uterine attachment and arterial remodeling. Motivated by our in vitro exposure data showing that BDE-47 inhibits CTB migration and invasion, we further investigated whether PBDE exposures in vivo were associated with reduced immunoreactivity of molecules that are typically upregulated during these processes. The association between decreased ITGA1 expression in endovascular CTBs and high levels of PBDE exposures was consistent with this hypothesis. However, we also observed a positive association between these exposures and enhanced immunoreactivity for CDH5 in invasive CTBs (interstitial and endovascular), for which there may be several possible explanations, given the large constellation of molecules (including transcription factors, adhesion molecules, and proteinases) and inhibitors that interact during placental invasion and vascular remodeling.

The exact mechanisms and molecular interactions during placentation remain unresolved, leaving many potential reasons for the discordant PBDE results between ITGA1 and CDH5. Compensatory mechanisms, other environmental contributors [45], developmental and localized timing of the expression of ITGA1/CDH5, and human variability may also influence the effect of PBDE exposure on the presence of ITGA1/CDH5 in invading populations of CTBs. For example, upregulated CDH5, which is an endothelial specific adhesion molecule expressed by CTBs as they migrate towards the uterus and translocate to the endothelial-lined maternal blood vessels [57], may be compensating for downregulated ITGA1, which is thought to help regulate the depth of trophoblast invasion [9]. PBDEs can also disrupt other pathways that regulate CTB differentiation, including hormone signaling, oxidative stress, inflammation, and placental developmental pathways [31, 33, 35, 40, 41], however, the direct interactions leading to increased CDH5 expression during CTB invasion is currently unknown and speculative in the context of environmental exposures. Moreover, we did not account for exposure to other chemicals such as Bisphenol-A (BPA), an endocrine disrupting compound that promotes trophoblast migration and disrupts placentation in mice by increasing the expression of invasion-promoting integrins (i.e.*,* ITGB2 and ITGA5) and metalloproteinases (i.e., MMP-2 and MMP-9) [58]. Finally, despite the relatively large sample size of our study, variability was limited since we could not always sample in every placental region. Nevertheless, while the molecular complexity of CTB invasion presents challenges for predicting outcomes of biomarker experiments in the context of the exposome, future work linking PBDE and other chemical exposures with well-studied molecular measures of placenta-mediated pregnancy complications (i.e., CDH5 and ITGA1) and other sensitive indicators of placental development and disease would strengthen epidemiologic research on chemical exposures and placenta-mediated adverse health outcomes.

As to the MMP1 analyses, the effects of in vitro PBDE exposures were analyzed at the RNA level [40] as compared to the antibody-based protein approach we employed. Lack of correlation between transcriptomic and proteomic data is now a well-recognized phenomenon [59] and the effects of acute (in vitro) vs. chronic (in vivo) PBDE exposures are not well understood. Moreover, subtle effects from chemical exposures are difficult to detect in smaller epidemiologic studies (due to substantial human variability that can diminish statistical power). Additional biomarker studies with environmental chemicals that may have stronger effects on placental development, such as BPA, per- and poly-fluorinated compounds (PFAS), and heavy metals, could potentially highlight the utility of MMP1 and other potential molecular biomarker candidates in future studies. Nevertheless, our use of high resolution and high precision measures can generate useful insights even in smaller epidemiologic studies, given the rigorous approach we took to directly measuring biological endpoints, linking molecular and morphologic biomarkers, and measuring exposures and outcomes within a critical developmental window.

Our additional analysis of matched maternal-fetal tissue samples revealed reasonably consistent patterns of association between molecular placental biomarkers and PBDE measurements across maternal and fetal tissues, with stronger PBDE associations observed in fetal liver samples. This may not be surprising, given the extreme interdependence of placental and fetal development during pregnancy, as well as the intricate coordination of hormones and molecular signaling pathways that drive biological changes and physiological adaptations between maternal, placental, and fetal compartments. Further efforts to identify possible mechanisms of placental PBDE toxicity relevant to fetal exposures are warranted.

Although we found no association between placental PBDE concentrations and the morphological endpoints we analyzed, our study is one of the first to characterize several important features of placental morphology during mid-gestation, a critical time of placentation about which current biological data is limited. Additionally, null associations are consistent with the fact that the morphological changes we examined are indicative of severe placental disease and damage, which may not be apparent until later in pregnancy. Given that typical PBDE exposures during pregnancy are not consistently associated with overt pregnancy complications, it is likely that these (and other environmental chemicals) have more subtle effects on placental tissues that are difficult to discern with the morphological features we selected. Identifying the most sensitive upstream physiological endpoints during mid-gestation that are relevant for placenta-mediated diseases would improve the utility of examining morphological features in future studies. Further integrating these physiological parameters with additional molecular and clinical measures that have been shown to improve diagnostic accuracy for complications like preeclampsia and fetal growth restriction (e.g., placental growth factor, fms-like tyrosine kinase-1, and advanced ultrasound techniques) [19] would also strengthen future research in this area. Moreover, efforts to identify circulating measures or imaging biomarkers that are more practical to measure in a larger sample would further advance the impact of studies conducted on placental tissues (such as ours) which are not accessible in intact pregnancies. Nevertheless, the use of primary human placental tissues is a strength of this study, as it enables direct correlation of the relationships examined and reduces uncertainties in extrapolation. As a complement to these studies, experimental investigations using primary cells, cell lines or animal models can be utilized to investigate relationships between exposure and placental dysfunction while controlling for many of the inherent challenges of observational studies (e.g., background exposures, gestational age and genetic variation, obtaining tissue).

In the time since we initiated this analysis, the application of new technologies for global profiling have emerged as a powerful way of answering important questions that are being raised about the effects of environmental chemical exposures on placental development. Many of these technologies could be applied to studies of similar design to ours in the future. For example, laser capture of specific trophoblast populations combined with global protein profiling by mass spectrometry could reveal interesting associations with environmental chemical exposures that are not biased toward molecules with well-studied roles in CTB differentiation and placental development. Also, discovering additional biomarkers of placental pathways (e.g., oxidative stress, inflammation, angiogenesis, and/or hormone signaling) that are perturbed by PBDE exposures could yield valuable insights into how these and other environmental chemicals impact pregnancy. Finally, given the explosive rate of placental development, it will be interesting to apply this approach across gestation to identify particularly important periods of vulnerability.

## Conclusions

We conducted a novel epidemiologic study using a rigorous approach for measuring biomarkers of PBDE exposure and placental development and disease with high resolution and precision. We examined multiple molecular indicators of placenta-mediated pregnancy complications that may also be sensitive indicators of maternal-fetal PBDE exposures. This novel approach can be applied more widely to examine additional molecular biomarkers of placental development and function (e.g., placental hormones, angiogenic factors, etc.) and/or other early indicators of pregnancy complications like preeclampsia (e.g., blood pressure) in epidemiologic studies of chemical exposures and adverse maternal-fetal health outcomes. Our findings indicate that placental biomarkers of development and disease could be useful barometers of exposure to PBDEs, a paradigm that could be extended to other environmental chemicals and placental stage-specific antigens.

## Supplementary information

**Additional file 1: Table S1.** Molecular and morphological biomarkers of placental disease and development during mid-gestation. **Table S2.** Primary antibodies used for immunolocalization assessments. **Table S3.** Molecular immunoreactivity and low/high PBDE exposure by placental region/cell type during mid-gestation (*n* = 62). **Table S4.** Detection frequencies for 19 PBDE congeners in matched samples of maternal serum, placenta, and fetal liver during mid-gestation (*n* = 130). **Table S5.** Correlation (unadjusted *p*-value)^*a*^ of molecular and morphological biomarkers during mid-gestation (*n* = 62). **Table S6.** Correlation (FDR estimate)^*a*^ of molecular and morphological placental biomarkers during mid-gestation (*n* = 62). **Table S7.** Correlation (unadjusted *p*-values and FDR)^*a*^ of placental biomarkers and wet-weight PBDE levels during mid-gestation (*n* = 62).

**Additional file 2: Figure S1.** Diagram of study framework illustrating approach to evaluating relationships between biomarkers of chemical exposure and potential biomarkers of placental development and disease. **Figure S2.** Immunoreactivity of ITGA1 at the maternal-fetal interface. Representative images of floating (**A-C**) and anchoring (**D-F**) villi, interstitial invading CTBs (CTBi) of the decidua (**G-I**), and endovascular CTBs (CTBe) lining a uterine artery (**J-L**). Tissue sections of 2nd trimester samples were immunostained for ITGA1 (green) and co-stained with anti-CK (trophoblast marker, red) and DAPI (nuclear dye, blue). Images represent typical profiles of ITGA1 expression. Bars = 100 μm. **Figure S3.** Immunoreactivity of CDH5 at the maternal-fetal interface. Representative images of floating (**A-C**) and anchoring (**D-F**) villi, interstitial invading CTBs (CTBi) of the decidua (**G-I**), and endovascular CTBs (CTBe) lining a uterine artery (**J-L**). Tissue sections of 2nd trimester samples were immunostained for ITGA1 (green) and co-stained with anti-CK (trophoblast marker, red) and DAPI (nuclear dye, blue). Images represent typical profile of CDH5 expression. Bars = 100 μm. **Figure S4.** Immunoreactivity of MMP1 at the maternal-fetal interface. Representative images of floating (**A-C**) and anchoring (**D-F**) villi, interstitial invading CTBs (CTBi) of the decidua (**G-I**), and endovascular CTBs (CTBe) lining a uterine artery (**J-L**). Tissue sections of 2nd trimester samples were immunostained for ITGA1 (green) and co-stained with anti-CK (trophoblast marker, red) and DAPI (nuclear dye, blue). Images represent typical profile of MMP1 expression. Bars = 100 μm. **Figure S5.** Representative morphological features of the maternalfetal interface. Representative images of morphological features that were scored in human placental biopsies: (**A**) perivillous fibrinoid deposition surrounding floating villi, (**B**) fibrinoid deposition, (**C**) leukocyte (white blood cell) infiltration of the basal plate, and (**D**) CTB remodeling of uterine arteries. Black arrows and dotted black lines indicate the feature of interest.

**Additional file 3.**

## Data Availability

The datasets used and/or analyzed during the current study are available from the corresponding author on reasonable request.

## Abbreviations

AV: Anchoring villi
BP: Basal plate
BMI: Body mass index
CDH5: Vascular endothelial-cadherin
CK: Cytokeratin
CTB: Cytotrophoblast
dAV: Distal anchoring villi
ECM: Extracellular matrix
eCTB: Endovascular CTB
EDC: Endocrine disrupting chemical
FV: Floating villi
H&E: Hematoxylin and Eosin
iCTB: Invasive/extravillous CTB
IF: Immunofluorescence
ITGA1: Integrin alpha-1
MDL: Method detection limit
MLE: Maximum likelihood estimation
MMP1: Metalloproteinase-1
MTBE: Methyl tert-butyl ether
pAV: Proximal anchoring villi
PBDE: Polybrominated diphenyl ethers
PFAS: Per- and polyfluorinated alkyl substances
SES: Socioeconomic status
STB: Syncytiotrophoblast
TH: Thyroid hormone
UCSF: University of California, San Francisco
US: United States
WBC: White blood cell
WOC: Women’s Options Center
