# Supplementary material for: Association of polybrominated diphenyl ether (PBDE) levels with biomarkers of placental development and disease during mid-gestation
Source: Environ Health. 2020 Jun 3;19:61. doi: 10.1186/s12940-020-00617-7 (PMC7268484; doi:10.1186/s12940-020-00617-7)
Supplement: Supplementary file 1 — Additional file 1: Table S1. Molecular and morphological biomarkers of placental disease and development during mid-gestation. Table S2. Primary antibodies used for immunolocalization assessments. Table S3. Molecular immunoreactivity and low/high PBDE exposure by placental region/cell type during mid-gestation (n = 62). Table S4. Detection frequencies for 19 PBDE congeners in matched samples of maternal serum, placenta, and fetal liver during mid-gestation (n = 130). Table S5. Correlation (unadjusted p-value)a of molecular and morphological biomarkers during mid-gestation (n = 62). Table S6. Correlation (FDR estimate)a of molecular and morphological placental biomarkers during mid-gestation (n = 62). Table S7. Correlation (unadjusted p-values and FDR)a of placental biomarkers and wet-weight PBDE levels during mid-gestation (n = 62). [file 12940_2020_617_MOESM1_ESM.docx]

**Supplemental Tables**

| Supplemental Table S1. Molecular and morphological biomarkers of placental disease and development during mid-gestation. | | | | |
| --- | --- | --- | --- | --- |
| Placental Biomarker | **Type** | **Definition** | **Role during Placentation** | **Potential Disease Associations** |
| ITGA1  *Integrin alpha-1* | Molecular | *Cell-ECM adhesion receptor*, Placenta-specific gene, cAMP-regulated. | Decrease during placentation may indicate reduced CTB invasion. | PE |
| CHD5  *Vascular endothelial-cadherin* | Molecular | *Cell-cell adhesion receptor*, Invasion, endovascular CTBs, blood vessel formation (angiogenesis). | Decrease during placentation may indicate less endovascular CTB invasion. | PE, other complications |
| MMP1 *Metalloproteinase-1* | Molecular | *Cell-ECM matrix degrading enzyme*, multiple functions (invasion, etc.). | Decrease during placentation may indicate reduced CTB cell migration/invasion. | PE, preterm birth |
| Fibrinoid Deposition  *Perivillous fibrinoid deposits in floating villi (fetal) and basal plate (maternal)* | Morphological | % Floating villi with any fibrinoid deposition (binary); % fibrinoid deposition in basal plate (area). | Increase during pregnancy may indicate placental stress. | PE |
| Leukocyte Deposition  *White blood cells in basal plate* | Morphological | Number of white blood cells per image. | Increase during pregnancy may indicate placental stress. | PE |
| CTB-modulated Blood Vessels  *Endovascular CTBs in basal plate* | Morphological | Spiral arteries with > 50% endovascular CTB modulation. | Decrease during pregnancy may indicate placental stress. | PE, other complications |
| CTB = Cytotrophoblast; ECM = Extracellular Matrix; PE = Preeclampsia.  *[Add citations: Fisher et al., 1989; Damsky et al., 1994, Librach et al., 1991, Zhou et al., 1997, Robinson et al., 2018]* | | | | |

| Supplemental Table S2. Primary antibodies used for immunolocalization assessments. | | | |
| --- | --- | --- | --- |
| Protein | Name | Company (#) | Host |
| MMP1 | Matrix metalloproteinase 1 | Abcam, ab38929 | Rb (polyclonal, 1:100) |
| ITGA1 | Integrin alpha 1 |  |  |
| CHD5 | Vascular endothelial cadherin |  |  |
| Cytokeratin | Cytoskeleton, TB-marker | 7Δ3, Damsky, 1992 | Rt (monoclonal, 1:100) |

| **Supplemental Table S3.** Molecular immunoreactivity and low/high PBDE exposure by placental region/cell type during mid-gestation (n=62). | | | | | | | | | | | | | | | | | | | | |
| --- | --- | --- | --- | --- | --- | --- | --- | --- | --- | --- | --- | --- | --- | --- | --- | --- | --- | --- | --- | --- |
|  |  | **ITGA1** | | | | | | |  | **CHD5** | | | | |  | **MMP1** | | | | |
| *Molecular Immunoreactivity* | | *Wet-weight*  *BDE-47^a^ No. (%)* | | | |  |  | *p-Value^b^* |  | *Wet-weight BDE-47^a^* | |  |  | *p-Value^b^* |  | *Wet-weight BDE-47^a^* | |  |  | *p-Value^b^* |
| ***Floating Villi*** | | *Low* | | *High* | | *Total* |  |  |  | *Low* | *High* | *Total* |  |  |  | *Low* | *High* | *Total* |  |  |
|  | <25% (-) | 29 | | 31 | | *60* | 100 |  |  | 25 | 28 | *53* | 88 |  |  | 9 | 13 | *22* | 37 |  |
|  | 25-75% (-/+) | 0 | | 0 | | *0* | 0 |  |  | 0 | 0 | *0* | 0 |  |  | 20 | 18 | *38* | 63 |  |
|  | >75% (+) | 0 | | 0 | | *0* | 0 |  |  | 4 | 3 | *7* | 12 |  |  | 0 | 0 | *0* | 0 |  |
|  | *Total* | *29* | | *31* | | *60* | *100* | *--* |  | *29* | *31* | *60* | 100 | *0.705* |  | *29* | *31* | *60* | *100* | *0.455* |
| ***Anchoring Villi (proximal)*** | | | |  | |  |  |  |  |  |  |  |  |  |  |  |  |  |  |  |
|  | <25% (-) | 24 | | 24 | | *48* | 92 |  |  | 6 | 5 | *11* | 21 |  |  | 1 | 0 | *1* | 2.0 |  |
|  | 25-75% (-/+) | 0 | | 2 | | *2* | 3.9 |  |  | 8 | 13 | *21* | 40 |  |  | *11* | *19* | *30* | 59 |  |
|  | >75% (+) | *1* | | *1* | | *2* | 3.9 |  |  | 11 | 10 | *21* | 40 |  |  | 11 | 9 | *20* | 39 |  |
|  | *Total* | *25* | | *27* | | *52* | *100* | *0.738* |  | *25* | *28* | *53* | 100 | *0.634* |  | *23* | *28* | *51* | *100* | *0.200* |
| ***Anchoring Villi (distal)*** | | |  | |  |  |  |  |  |  |  |  |  |  |  |  |  |  |  |  |
|  | <25% (-) | 18 | | 20 | | *38* | 73 |  |  | *6* | *3* | *9* | 17 |  |  | 0 | 0 | *0* | 0 |  |
|  | 25-75% (-/+) | 2 | | 1 | | *3* | 5.8 |  |  | 10 | 14 | *24* | 45 |  |  | 10 | 15 | *25* | 49 |  |
|  | >75% (+) | 5 | | 6 | | *11* | 21 |  |  | 9 | 11 | *20* | 38 |  |  | *13* | *13* | *26* | 51 |  |
|  | *Total* | *25* | | *27* | | *52* | *100* | *0.895* |  | *25* | *28* | *53* | 100 | *0.514* |  | *23* | *28* | *51* | *100* | *0.566* |
| ***Interstitial CTB*** | |  | |  | |  |  |  |  |  |  |  |  |  |  |  |  |  |  |  |
|  | <25% (-) | 2 | | 4 | | *6* | 10 |  |  | 7 | 1 | *8* | 13 |  |  | 2 | 0 | *2* | 3.3 |  |
|  | 25-75% (-/+) | 15 | | 9 | | *24* | 40 |  |  | 4 | 10 | *14* | 23 |  |  | 11 | 12 | *23* | 38 |  |
|  | >75% (+) | 12 | | 18 | | *30* | 50 |  |  | 18 | 20 | *38* | 63 |  |  | 16 | 19 | *35* | 58 |  |
|  | *Total* | *29* | | *31* | | *60* | *100* | *0.197* |  | *29* | *31* | *60* | 100 | ***0.023**** |  | *29* | *31* | *60* | *100* | *0.476* |
| ***Endovascular CTB*** | |  | |  | |  |  |  |  |  |  |  |  |  |  |  |  |  |  |  |
|  | <25% (-) | 1 (3.4) | | 5 (17) | | *6 (21)* | 21 |  |  | 6 | 2 | *8* | 19 |  |  | 6 | 5 | *11* | 31 |  |
|  | 25-75% (-/+) | 3 (10) | | 2 (6.9) | | *5 (17)* | 17 |  |  | 1 | 2 | *3* | 7.1 |  |  | 10 | 10 | *20* | 56 |  |
|  | >75% (+) | 14 (48) | | 4 (14) | | *18 (62)* | 62 |  |  | 18 | 13 | *31* | 74 |  |  | 3 | 2 | *5* | 14 |  |
|  | *Total* | *18* | | *11* | | *29* | *100* | ***0.022**** |  | *25* | *17* | *42* | 100 | *0.497* |  | *19* | *17* | *36* | *100* | *1.00* |
| CTB = Cytotrophoblast. * *p* <0.05.  *^a^* Low/high PBDE exposure groups divided at the median.  *^b^* *P-Values* from Fisher's exact test of independence among categorical groups. | | | | | | | | | | | | | | | | | | | | |

| **Supplemental Table S4.** Detection frequencies for 19 PBDE congeners in matched samples of maternal serum, placenta, and fetal liver during mid-gestation (n=130). | | | | | | | | | | | | |
| --- | --- | --- | --- | --- | --- | --- | --- | --- | --- | --- | --- | --- |
|  | **Fetal Liver** | | | | **Placenta** | | | | **Maternal Serum** | | | |
| *Congener* | *MDL (ng/g)* | *Non-report†* | *N > MDL* | *% > MDL* | *MDL (ng/g)* | *Non-report†* | *N > MDL* | *% > MDL* | *MDL (ng/ml)* | *Non-report†* | *N > MDL* | *% > MDL* |
| BDE-17 | 0.008 | 0 | 2 | 1.5 | 0.004 | 1 | 0 | 0 | 0.02 | 0 | 0 | 0 |
| BDE-28 | 0.008 | 0 | 86 | 66 | 0.006 | 1 | 82 | 64 | 0.02 | 0 | 21 | 16 |
| BDE-47 | 0.042 | 0 | 128 | 99 | 0.017 | 0 | 130 | 100 | 0.04 | 0 | 128 | 99 |
| BDE-66 | 0.008 | 0 | 2 | 1.5 | 0.004 | 0 | 0 | 0 | 0.02 | 0 | 0 | 0 |
| BDE-85 | 0.008 | 0 | 10 | 7.7 | 0.004 | 0 | 4 | 3.1 | 0.02 | 0 | 2 | 1.5 |
| BDE-99 | 0.018 | 0 | 125 | 96 | 0.009 | 0 | 129 | 99 | 0.03 | 0 | 111 | 85 |
| BDE-100 | 0.009 | 0 | 123 | 95 | 0.004 | 0 | 130 | 100 | 0.02 | 0 | 101 | 78 |
| BDE-153 | 0.016 | 0 | 114 | 88 | 0.009 | 0 | 120 | 92 | 0.03 | 0 | 93 | 72 |
| BDE-154 | 0.016 | 0 | 4 | 3.1 | 0.008 | 0 | 5 | 3.8 | 0.03 | 0 | 0 | 0 |
| BDE-183 | 0.016 | 0 | 0 | 0 | 0.008 | 0 | 4 | 3.1 | 0.03 | 0 | 1 | 0.8 |
| BDE-196 | 0.016 | 1 | 0 | 0 | 0.008 | 0 | 2 | 1.5 | 0.03 | 0 | 0 | 0 |
| BDE-197 | 0.016 | 1 | 12 | 9.3 | 0.008 | 0 | 11 | 8.5 | 0.03 | 0 | 2 | 1.5 |
| BDE-201 | 0.016 | 1 | 0 | 0 | 0.008 | 0 | 0 | 0 | 0.03 | 0 | 0 | 0 |
| BDE-202 | 0.016 | 1 | 0 | 0 | 0.008 | 1 | 0 | 0 | 0.03 | 0 | 0 | 0 |
| BDE-203 | 0.016 | 1 | 0 | 0 | 0.008 | 0 | 2 | 1.5 | 0.03 | 0 | 0 | 0 |
| BDE-206 | 0.021 | 12 | 0 | 0 | 0.013 | 2 | 2 | 1.6 | 0.04 | 1 | 0 | 0 |
| BDE-207 | 0.021 | 12 | 10 | 8.5 | 0.014 | 2 | 11 | 8.6 | 0.04 | 1 | 1 | 0.8 |
| BDE-208 | 0.021 | 12 | 1 | 0.8 | 0.016 | 2 | 2 | 1.6 | 0.04 | 1 | 0 | 0 |
| BDE-209 | 0.200 | 38 | 8 | 8.7 | 0.066 | 45 | 9 | 11 | 0.10 | 13 | 5 | 4.3 |
| MDL = Method detection limit. Green color indicates > 50% detection frequency. | | | | | | | | | | | | |
| † Not reported due to sample loss or surrogate failure. Not included in detection frequency calculation. | | | | | | | | | | | | |

| **Supplemental Table S5.** Correlation (unadjusted p-value)*^a^* of molecular and morphological biomarkers during mid-gestation (n=62). | | | | | | | | | | | | | | | | | | | |
| --- | --- | --- | --- | --- | --- | --- | --- | --- | --- | --- | --- | --- | --- | --- | --- | --- | --- | --- | --- |
|  |  | **MMP1** | | | | | **ITGA1** | | | | **CHD5** | | | | | **WBC** | **fvFD** | **bpFD** | **mBV** |
|  |  | *FV* | *pAV* | *dAV* | *iCTB* | *eCTB* | *pAV* | *dAV* | *iCTB* | *eCTB* | *FV* | *pAV* | *dAV* | *iCTB* | *eCTB* |  |  |  |  |
| **MMP1** | *FV* | **1.00 (<0.001)** | 0.07 (0.63) | **0.34 (0.02)** | **0.26 (0.04)** | 0.18 (0.28) | -0.20 (0.16) | 0.14 (0.29) | 0.12 (0.36) | 0.28 (0.12) | -0.15 (0.24) | 0.22 (0.09) | -0.04 (0.74) | -0.03 (0.83) | -0.12 (0.45) | 0.02 (0.84) | **-0.29 (0.01)** | -0.12 (0.29) | -0.09 (0.54) |
|  | *pAV* | 0.07 (0.63) | **1.00 (<0.001)** | **0.42 (<0.001)** | 0.18 (0.21) | 0.29 (0.10) | 0.07 (0.62) | -0.14 (0.32) | -0.10 (0.48) | **0.45 (0.02)** | 0.09 (0.54) | -0.11 (0.41) | -0.15 (0.28) | 0.42 (<0.001) | 0.18 (0.29) | -0.09 (0.43) | **0.22 (0.06)** | 0.20 (0.09) | 0.16 (0.28) |
|  | *dAV* | **0.34 (0.02)** | **0.42 (<0.001)** | **1.00 (<0.001)** | **0.46 (<0.001)** | 0.20 (0.26) | -0.01 (0.93) | **0.31 (0.03)** | 0.10 (0.46) | **0.38 (0.06)** | **-0.25 (0.08)** | 0.19 (0.18) | 0.17 (0.23) | 0.12 (0.37) | 0.14 (0.42) | **-0.27 (0.02)** | **-0.29 (0.01)** | -0.20 (0.10) | 0.12 (0.43) |
|  | *iCTB* | **0.26 (0.04)** | 0.18 (0.21) | **0.46 (<0.001)** | **1.00 (<0.001)** | **0.32 (0.05)** | **0.23 (0.09)** | **0.32 (0.02)** | **0.23 (0.07)** | 0.20 (0.26) | -0.04 (0.75) | 0.25 (0.05) | 0.10 (0.46) | -0.01 (0.92) | -0.19 (0.21) | **-0.26 (0.02)** | -0.01 (0.90) | 0.07 (0.54) | -0.12 (0.41) |
|  | *eCTB* | 0.18 (0.28) | 0.29 (0.10) | 0.20 (0.26) | **0.32 (0.05)** | **1.00 (<0.001)** | 0.07 (0.66) | -0.02 (0.92) | 0.05 (0.77) | -0.07 (0.75) | -0.23 (0.16) | -0.06 (0.72) | 0.18 (0.29) | 0.45 (<0.001) | 0.14 (0.42) | 0.02 (0.89) | 0.04 (0.74) | 0.00 (1.00) | 0.26 (0.12) |
| **ITGA1** | *pAV* | -0.20 (0.16) | 0.07 (0.62) | -0.01 (0.93) | **0.23 (0.09)** | 0.07 (0.66) | **1.00 (<0.001)** | **0.46 (<0.001)** | 0.14 (0.29) | 0.15 (0.45) | -0.10 (0.46) | 0.12 (0.39) | 0.12 (0.39) | 0.20 (0.13) | -0.05 (0.75) | 0.00 (0.99) | 0.12 (0.29) | 0.10 (0.40) | -0.06 (0.71) |
|  | *dAV* | 0.14 (0.29) | -0.14 (0.32) | **0.31 (0.03)** | **0.32 (0.02)** | -0.02 (0.92) | **0.46 (<0.001)** | 1.00 (<0.001) | 0.42 (<0.001) | 0.31 (0.11) | -0.21 (0.12) | **0.27 (0.04)** | 0.19 (0.17) | 0.13 (0.31) | 0.14 (0.41) | -0.21 (0.07) | -0.24 (0.04) | -0.24 (0.03) | -0.10 (0.49) |
|  | *iCTB* | 0.12 (0.36) | -0.10 (0.48) | 0.10 (0.46) | **0.23 (0.07)** | 0.05 (0.77) | 0.14 (0.29) | 0.42 (<0.001) | 1.00 (<0.001) | **0.31 (0.07)** | -0.01 (0.94) | 0.17 (0.19) | 0.08 (0.56) | 0.14 (0.26) | 0.01 (0.92) | -0.10 (0.32) | 0.00 (0.99) | 0.05 (0.63) | -0.02 (0.89) |
|  | *eCTB* | 0.28 (0.12) | **0.45 (0.02)** | **0.38 (0.06)** | 0.20 (0.26) | -0.07 (0.75) | 0.15 (0.45) | 0.31 (0.11) | **0.31 (0.07)** | **1.00 (<0.001)** | -0.16 (0.38) | -0.24 (0.22) | -0.15 (0.43) | 0.14 (0.42) | 0.35 (0.07) | 0.03 (0.85) | 0.14 (0.35) | 0.21 (0.17) | -0.04 (0.82) |
| **CHD5** | *FV* | -0.15 (0.24) | 0.09 (0.54) | -0.25 (0.08) | -0.04 (0.75) | -0.23 (0.16) | -0.10 (0.46) | -0.21 (0.12) | -0.01 (0.94) | -0.16 (0.38) | **1.00 (<0.001)** | 0.07 (0.61) | -0.02 (0.87) | -0.04 (0.75) | -0.06 (0.70) | -0.14 (0.21) | -0.04 (0.72) | 0.00 (0.98) | 0.04 (0.80) |
|  | *pAV* | **0.22 (0.09)** | -0.11 (0.41) | 0.19 (0.18) | 0.25 (0.05) | -0.06 (0.72) | 0.12 (0.39) | **0.27 (0.04)** | 0.17 (0.19) | -0.24 (0.22) | 0.07 (0.61) | **1.00 (<0.001)** | **0.64 (<0.001)** | 0.00 (0.98) | -0.03 (0.83) | -0.23 (0.04) | -0.14 (0.20) | -0.28 (0.01) | 0.00 (0.99) |
|  | *dAV* | -0.04 (0.74) | -0.15 (0.28) | 0.17 (0.23) | 0.10 (0.46) | 0.18 (0.29) | 0.12 (0.39) | 0.19 (0.17) | 0.08 (0.56) | -0.15 (0.43) | -0.02 (0.87) | **0.64 (<0.001)** | **1.00 (<0.001)** | 0.23 (0.07) | 0.24 (0.13) | -0.26 (0.02) | -0.04 (0.74) | -0.18 (0.10) | **0.25 (0.09)** |
|  | *iCTB* | -0.03 (0.83) | **0.42 (<0.001)** | 0.12 (0.37) | -0.01 (0.92) | **0.45 (<0.001)** | 0.20 (0.13) | 0.13 (0.31) | 0.14 (0.26) | 0.14 (0.42) | -0.04 (0.75) | 0.00 (0.98) | 0.23 (0.07) | **1.00 (<0.001)** | **0.49 (<0.001)** | -0.12 (0.23) | 0.12 (0.26) | 0.05 (0.61) | **0.43 (<0.001)** |
|  | *eCTB* | -0.12 (0.45) | 0.18 (0.29) | 0.14 (0.42) | -0.19 (0.21) | 0.14 (0.42) | -0.05 (0.75) | 0.14 (0.41) | 0.01 (0.92) | **0.35 (0.07)** | -0.06 (0.70) | -0.03 (0.83) | 0.24 (0.13) | **0.49 (<0.001)** | **1.00 (<0.001)** | -0.01 (0.94) | 0.15 (0.25) | -0.01 (0.93) | 0.42 (0.01) |
| **WBC** | | 0.02 (0.84) | -0.09 (0.43) | **-0.27 (0.02)** | **-0.26 (0.02)** | 0.02 (0.89) | 0.00 (0.99) | **-0.21 (0.07)** | -0.10 (0.32) | 0.03 (0.85) | -0.14 (0.21) | **-0.23 (0.04)** | **-0.26 (0.02)** | -0.12 (0.23) | -0.01 (0.94) | **1.00 (<0.001)** | 0.06 (0.46) | 0.08 (0.36) | -0.13 (0.27) |
| **fvFD** | | **-0.29 (0.01)** | **0.22 (0.06)** | **-0.29 (0.01)** | -0.01 (0.90) | 0.04 (0.74) | 0.12 (0.29) | **-0.24 (0.04)** | 0.00 (0.99) | 0.14 (0.35) | -0.04 (0.72) | -0.14 (0.20) | -0.04 (0.74) | 0.12 (0.26) | 0.15 (0.25) | 0.06 (0.46) | **1.00 (<0.001)** | **0.24 (0.01)** | 0.08 (0.51) |
| **bpFD** | | -0.12 (0.29) | **0.20 (0.09)** | -0.20 (0.10) | 0.07 (0.54) | 0.00 (1.00) | 0.10 (0.40) | **-0.24 (0.03)** | 0.05 (0.63) | 0.21 (0.17) | 0.00 (0.98) | -0.28 **(0.01)** | -0.18 (0.10) | 0.05 (0.61) | -0.01 (0.93) | 0.08 (0.36) | **0.24 (0.01)** | **1.00 (<0.001)** | -0.12 (0.31) |
| **mBV** | | -0.09 (0.54) | 0.16 (0.28) | 0.12 (0.43) | -0.12 (0.41) | 0.26 (0.12) | -0.06 (0.71) | -0.10 (0.49) | -0.02 (0.89) | -0.04 (0.82) | 0.04 (0.80) | 0.00 (0.99) | **0.25 (0.09)** | **0.43 (<0.001)** | **0.42 (0.01)** | -0.13 (0.27) | 0.08 (0.51) | -0.12 (0.31) | **1.00 (<0.001)** |
| dAV = Anchoring Villi (distal); pAV = Anchoring Villi (proximal); eCTB = Endovascular CTB; iCTB = Interstitial CTB; FV = Floating Villi (bilayer); bpFD = % Fibrinoid Deposition in Basal Plate; fvFD = % Floating Villi with Fibrinoid Deposition; mBV = % CTB-modulated blood vessels. WBC = White blood cell count.  *^a^* Rank order correlation assessed with Kendall’s Tau Correlation Coefficient (unadjusted for multiple comparisons). *P*-values < 0.10 bolded. | | | | | | | | | | | | | | | | | | | |

| **Supplemental Table S6.** Correlation (FDR estimate)*^a^* of molecular and morphological placental biomarkers during mid-gestation (n=62). | | | | | | | | | | | | | | | | | | | |
| --- | --- | --- | --- | --- | --- | --- | --- | --- | --- | --- | --- | --- | --- | --- | --- | --- | --- | --- | --- |
|  |  | **MMP1** | | | | | **ITGA1** | | | | **CHD5** | | | | | **WBC** | **fvFD** | **bpFD** | **mBV** |
|  |  | *FV* | *pAV* | *dAV* | *iCTB* | *eCTB* | *pAV* | *dAV* | *iCTB* | *eCTB* | *FV* | *pAV* | *dAV* | *iCTB* | *eCTB* |  |  |  |  |
| **MMP1** | *FV* | **1.00 (<0.001)** | 0.07 (0.63) | **0.34 (0.06)** | 0.26 (0.13) | 0.18 (0.57) | -0.20 (0.58) | 0.14 (0.38) | 0.12 (0.71) | 0.28 (0.30) | -0.15 (0.71) | 0.22 (0.23) | -0.04 (0.79) | -0.03 (0.93) | -0.12 (0.67) | 0.02 (0.99) | **-0.29 (0.05)** | -0.12 (0.56) | -0.09 (0.75) |
|  | *pAV* | 0.07 (0.76) | **1.00 (<0.001)** | **0.42 (0.02)** | 0.18 (0.34) | 0.29 (0.43) | 0.07 (0.85) | -0.14 (0.38) | -0.10 (0.79) | 0.45 (0.22) | 0.09 (0.96) | -0.11 (0.57) | -0.15 (0.47) | **0.42 (0.01)** | 0.18 (0.65) | -0.09 (0.64) | 0.22 (0.17) | 0.20 (0.26) | 0.16 (0.70) |
|  | *dAV* | **0.34 (0.09)** | **0.42 (0.02)** | **1.00 (<0.001)** | **0.46 (0.01)** | 0.20 (0.57) | -0.01 (0.98) | **0.31 (0.09)** | 0.10 (0.79) | 0.38 (0.26) | -0.25 (0.69) | 0.19 (0.35) | 0.17 (0.47) | 0.12 (0.56) | 0.14 (0.67) | -0.27 (0.11) | -0.29 (0.06) | -0.20 (0.26) | 0.12 (0.75) |
|  | *iCTB* | 0.26 (0.19) | 0.18 (0.47) | **0.46 (0.01)** | **1.00 (<0.001)** | 0.32 (0.29) | 0.23 (0.55) | 0.32 (0.08) | 0.23 (0.33) | 0.20 (0.47) | -0.04 (0.96) | 0.25 (0.15) | 0.10 (0.60) | -0.01 (0.97) | -0.19 (0.62) | -0.26 (0.11) | -0.01 (0.96) | 0.07 (0.75) | -0.12 (0.75) |
|  | *eCTB* | 0.18 (0.48) | 0.29 (0.26) | 0.20 (0.36) | 0.32 (0.13) | **1.00 (<0.001)** | 0.07 (0.85) | -0.02 (0.92) | 0.05 (0.99) | -0.07 (0.84) | -0.23 (0.71) | -0.06 (0.87) | 0.18 (0.47) | 0.45 (0.01) | 0.14 (0.67) | 0.02 (0.99) | 0.04 (0.84) | 0.00 (1.00) | 0.26 (0.43) |
| **ITGA1** | *pAV* | -0.20 (0.41) | 0.07 (0.63) | -0.01 (0.93) | 0.23 (0.18) | 0.07 (0.92) | **1.00 (<0.001)** | **0.46 (0.01)** | 0.14 (0.71) | 0.15 (0.54) | -0.10 (0.96) | 0.12 (0.57) | 0.12 (0.58) | 0.20 (0.33) | -0.05 (0.94) | 0.00 (0.99) | 0.12 (0.52) | 0.10 (0.60) | -0.06 (0.91) |
|  | *dAV* | 0.14 (0.48) | -0.14 (0.47) | **0.31 (0.09)** | 0.32 **(0.08)** | -0.02 (0.97) | 0.46 (0.01) | **1.00 (<0.001)** | **0.42 (0.01)** | 0.31 (0.30) | -0.21 (0.71) | 0.27 (0.15) | 0.19 (0.37) | 0.13 (0.51) | 0.14 (0.67) | -0.21 (0.20) | -0.24 (0.13) | -0.24 (0.15) | -0.10 (0.75) |
|  | *iCTB* | 0.12 (0.54) | -0.10 (0.58) | 0.10 (0.49) | 0.23 (0.15) | 0.05 (0.92) | 0.14 (0.69) | **0.42 (0.01)** | **1.00 (<0.001)** | 0.31 (0.26) | -0.01 (0.98) | 0.17 (0.35) | 0.08 (0.67) | 0.14 (0.46) | 0.01 (0.94) | -0.10 (0.57) | 0.00 (0.99) | 0.05 (0.75) | -0.02 (0.95) |
|  | *eCTB* | 0.28 (0.35) | 0.45 (0.11) | 0.38 (0.14) | 0.20 (0.39) | -0.07 (0.92) | 0.15 (0.69) | 0.31 (0.20) | 0.31 (0.33) | **1.00 (<0.001)** | -0.16 (0.96) | -0.24 (0.37) | -0.15 (0.60) | 0.14 (0.58) | 0.35 (0.30) | 0.03 (0.99) | 0.14 (0.57) | 0.21 (0.38) | -0.04 (0.92) |
| **CDH5** | *FV* | -0.15 (0.48) | 0.09 (0.61) | -0.25 (0.15) | -0.04 (0.84) | -0.23 (0.48) | -0.10 (0.69) | -0.21 (0.20) | -0.01 (0.99) | -0.16 (0.54) | **1.00 (<0.001)** | 0.07 (0.78) | -0.02 (0.87) | -0.04 (0.90) | -0.06 (0.94) | -0.14 (0.52) | -0.04 (0.84) | 0.00 (1.00) | 0.04 (0.92) |
|  | *pAV* | 0.22 (0.32) | -0.11 (0.56) | 0.19 (0.29) | 0.25 (0.13) | -0.06 (0.92) | 0.12 (0.69) | 0.27 (0.10) | 0.17 (0.68) | -0.24 (0.45) | 0.07 (0.96) | **1.00 (<0.001)** | 0.64 (<0.001) | 0.00 (0.98) | -0.03 (0.94) | -0.23 (0.13) | -0.14 (0.51) | -0.28 (0.06) | 0.00 (0.99) |
|  | *dAV* | -0.04 (0.84) | -0.15 (0.47) | 0.17 (0.35) | 0.10 (0.60) | 0.18 (0.57) | 0.12 (0.69) | 0.19 (0.25) | 0.08 (0.83) | -0.15 (0.54) | -0.02 (0.98) | **0.64 (<0.001)** | **1.00 (<0.001)** | 0.23 (0.21) | 0.24 (0.48) | -0.26 (0.11) | -0.04 (0.84) | -0.18 (0.26) | 0.25 (0.40) |
|  | *iCTB* | -0.03 (0.84) | **0.42 (0.01)** | 0.12 (0.48) | -0.01 (0.92) | **0.45 (0.04)** | 0.20 (0.57) | 0.13 (0.38) | 0.14 (0.71) | 0.14 (0.54) | -0.04 (0.96) | 0.00 (0.99) | 0.23 (0.31) | **1.00 (<0.001)** | **0.49 (0.01)** | -0.12 (0.52) | 0.12 (0.52) | 0.05 (0.75) | 0.43 (0.02) |
|  | *eCTB* | -0.12 (0.62) | 0.18 (0.47) | 0.14 (0.48) | -0.19 (0.34) | 0.14 (0.76) | -0.05 (0.85) | 0.14 (0.46) | 0.01 (0.99) | 0.35 (0.26) | -0.06 (0.96) | -0.03 (0.94) | 0.24 (0.34) | 0.49 (0.01) | **1.00 (<0.001)** | -0.01 (0.99) | 0.15 (0.52) | -0.01 (1.00) | 0.42 (0.05) |
| **WBC** | | 0.02 (0.84) | -0.09 (0.56) | **-0.27 (0.07)** | -0.26 (0.08) | 0.02 (0.97) | 0.00 (0.99) | -0.21 (0.13) | -0.10 (0.71) | 0.03 (0.85) | -0.14 (0.71) | -0.23 (0.15) | -0.26 (0.12) | -0.12 (0.46) | -0.01 (0.94) | **1.00 (<0.001)** | 0.06 (0.69) | 0.08 (0.59) | -0.13 (0.70) |
| **fvFD** | | **-0.29 (0.07)** | 0.22 (0.20) | -0.29 (0.06) | -0.01 (0.92) | 0.04 (0.92) | 0.12 (0.69) | -0.24 (0.09) | 0.00 (0.99) | 0.14 (0.54) | -0.04 (0.96) | -0.14 (0.35) | -0.04 (0.79) | 0.12 (0.46) | 0.15 (0.63) | 0.06 (0.64) | **1.00 (<0.001)** | **0.24 (0.06)** | 0.08 (0.75) |
| **bpFD** | | -0.12 (0.48) | 0.20 (0.26) | -0.20 (0.17) | 0.07 (0.65) | 0.00 (1.00) | 0.10 (0.69) | -0.24 (0.09) | 0.05 (0.87) | 0.21 (0.38) | 0.00 (0.98) | **-0.28 (0.06)** | -0.18 (0.31) | 0.05 (0.79) | -0.01 (0.94) | 0.08 (0.59) | **0.24 (0.05)** | **1.00 (<0.001)** | -0.12 (0.70) |
| **mBV** | | -0.09 (0.69) | 0.16 (0.47) | 0.12 (0.48) | -0.12 (0.56) | 0.26 (0.43) | -0.06 (0.85) | -0.10 (0.52) | -0.02 (0.99) | -0.04 (0.85) | 0.04 (0.96) | 0.00 (0.99) | 0.25 (0.31) | **0.43 (0.01)** | **0.42 (0.05)** | -0.13 (0.53) | 0.08 (0.70) | -0.12 (0.56) | **1.00 (<0.001)** |
| dAV = Anchoring Villi (distal); pAV = Anchoring Villi (proximal); eCTB = Endovascular CTB; iCTB = Interstitial CTB; FV = Floating Villi (bilayer); bpFD = % Fibrinoid Deposition in Basal Plate; fvFD = % Floating Villi with Fibrinoid Deposition; mBV = % CTB-modulated Blood Vessels. WBC = White blood cell count.  *^a^* Rank order correlation assessed using Kendall’s Tau Correlation Coefficient. False discovery rate (FDR) estimated from multiple comparisons adjustment. *P*-values and FDR < 0.10 bolded. | | | | | | | | | | | | | | | | | | | |

| **Supplemental Table S7.** Correlation (unadjusted p-values and FDR)*^a^* of placental biomarkers and wet-weight PBDE levels during mid-gestation (n=62). | | | | | | | | | | | | | |
| --- | --- | --- | --- | --- | --- | --- | --- | --- | --- | --- | --- | --- | --- |
|  |  | *BDE-28 (ng/g)* | | *BDE-47 (ng/g)* | | *BDE-99 (ng/g)* | | *BDE-100 (ng/g)* | | *BDE-153 (ng/g)* | | ***∑*** *PBDE4 (ng/g)* | |
|  |  | **Unadjusted** | **FDR** | **Unadjusted** | **FDR** | **Unadjusted** | **FDR** | **Unadjusted** | **FDR** | **Unadjusted** | **FDR** | **Unadjusted** | **FDR** |
| MMP1 | FV | 0.08 (0.25) | 1.00 | 0.10 (0.18) | 1.00 | 0.12 (0.12) | 1.00 | 0.07 (0.34) | 1.00 | 0.10 (0.19) | 1.00 | 0.11 (0.15) | 1.00 |
|  | pAV | 0.08 (0.33) | 1.00 | -0.04 (0.67) | 1.00 | -0.12 (0.15) | 1.00 | -0.03 (0.73) | 1.00 | -0.04 (0.61) | 1.00 | -0.05 (0.51) | 1.00 |
|  | dAV | 0.07 (0.39) | 1.00 | 0.02 (0.80) | 1.00 | 0.03 (0.74) | 1.00 | 0.00 (0.98) | 1.00 | 0.00 (0.96) | 1.00 | 0.01 (0.90) | 1.00 |
|  | iCTB | **0.18 (0.01)** | **0.24** | 0.10 (0.20) | 1.00 | 0.08 (0.30) | 1.00 | 0.10 (0.18) | 1.00 | 0.12 (0.11) | 1.00 | 0.10 (0.18) | 1.00 |
|  | eCTB | 0.07 (0.52) | 1.00 | 0.02 (0.88) | 1.00 | -0.03 (0.81) | 1.00 | -0.01 (0.95) | 1.00 | -0.11 (0.28) | 1.00 | -0.01 (0.93) | 1.00 |
| ITGA1 | pAV | 0.01 (0.83) | 1.00 | 0.03 (0.57) | 1.00 | 0.02 (0.74) | 1.00 | 0.01 (0.80) | 1.00 | 0.06 (0.17) | 1.00 | 0.03 (0.53) | 1.00 |
|  | dAV | 0.07 (0.33) | 1.00 | 0.01 (0.87) | 1.00 | 0.04 (0.57) | 1.00 | -0.02 (0.84) | 1.00 | 0.09 (0.24) | 1.00 | 0.03 (0.71) | 1.00 |
|  | iCTB | 0.10 (0.20) | 1.00 | 0.05 (0.54) | 1.00 | 0.05 (0.52) | 1.00 | 0.02 (0.77) | 1.00 | 0.09 (0.28) | 1.00 | 0.05 (0.56) | 1.00 |
|  | eCTB | -0.08 (0.45) | 1.00 | **-0.21 (0.07)** | 1.00 | **-0.20 (0.08)** | 1.00 | **-0.20 (0.08)** | 1.00 | -0.19 (0.10) | 1.00 | **-0.25 (0.03)** | **0.50** |
| CHD5 | FV | -0.03 (0.55) | 1.00 | -0.05 (0.31) | 1.00 | -0.07 (0.13) | 1.00 | -0.03 (0.61) | 1.00 | -0.01 (0.91) | 1.00 | -0.04 (0.45) | 1.00 |
|  | pAV | -0.02 (0.87) | 1.00 | -0.02 (0.80) | 1.00 | 0.00 (1.00) | 1.00 | -0.03 (0.77) | 1.00 | 0.07 (0.45) | 1.00 | 0.02 (0.83) | 1.00 |
|  | dAV | -0.06 (0.48) | 1.00 | -0.01 (0.90) | 1.00 | 0.01 (0.87) | 1.00 | -0.03 (0.72) | 1.00 | 0.01 (0.96) | 1.00 | 0.01 (0.93) | 1.00 |
|  | iCTB | 0.05 (0.49) | 1.00 | 0.03 (0.66) | 1.00 | 0.01 (0.92) | 1.00 | -0.01 (0.90) | 1.00 | -0.06 (0.41) | 1.00 | 0.00 (0.96) | 1.00 |
|  | eCTB | 0.01 (0.93) | 1.00 | 0.05 (0.53) | 1.00 | 0.03 (0.76) | 1.00 | -0.01 (0.89) | 1.00 | **-0.15 (0.07)** | 1.00 | 0.00 (0.98) | 1.00 |
| WBC | | -0.10 (0.25) | 1.00 | -0.09 (0.29) | 1.00 | -0.10 (0.26) | 1.00 | -0.11 (0.21) | 1.00 | -0.13 (0.15) | 1.00 | -0.12 (0.18) | 1.00 |
| fvFD | | -0.03 (0.76) | 1.00 | -0.07 (0.46) | 1.00 | **-0.15 (0.09)** | 1.00 | -0.08 (0.37) | 1.00 | -0.10 (0.25) | 1.00 | -0.10 (0.27) | 1.00 |
| bpFD | | 0.03 (0.72) | 1.00 | -0.02 (0.85) | 1.00 | -0.09 (0.33) | 1.00 | -0.02 (0.78) | 1.00 | 0.00 (1.00) | 1.00 | -0.04 (0.64) | 1.00 |
| mBV | | 0.08 (0.47) | 1.00 | 0.02 (0.84) | 1.00 | 0.00 (0.98) | 1.00 | 0.08 (0.51) | 1.00 | -0.04 (0.75) | 1.00 | 0.00 (1.00) | 1.00 |
| dAV = Anchoring Villi (distal); pAV = Anchoring Villi (proximal); eCTB = Endovascular CTB; iCTB = Interstitial CTB; FV = Floating Villi (bilayer); bpFD = % Fibrinoid Deposition in Basal Plate; fvFD = % Floating Villi with Fibrinoid Deposition; mBV = % CTB-modulated blood vessels. WBC = White blood cell count.  *^a^* Rank order correlation assessed with Kendall’s Tau Correlation Coefficient. False discovery rate (FDR) estimated from multiple comparisons adjustment using the Benjamini and Hochberg method (Benjamini and Hochberg, 1995). P-values < 0.10 and FDR estimates < 1.0 bolded. Similar pattern of results observed for lipid-adjusted BDE levels and were thus not reported. | | | | | | | | | | | | | |
