# Supplementary material for: Association of polybrominated diphenyl ether (PBDE) levels with biomarkers of placental development and disease during mid-gestation
Source: Environ Health. 2020 Jun 3;19:61. doi: 10.1186/s12940-020-00617-7 (PMC7268484; doi:10.1186/s12940-020-00617-7)

Supplemental Figures

Supplemental Figure S1. Diagram of study framework illustrating approach to evaluating relationships between biomarkers of chemical exposure and potential biomarkers of placental development and disease.

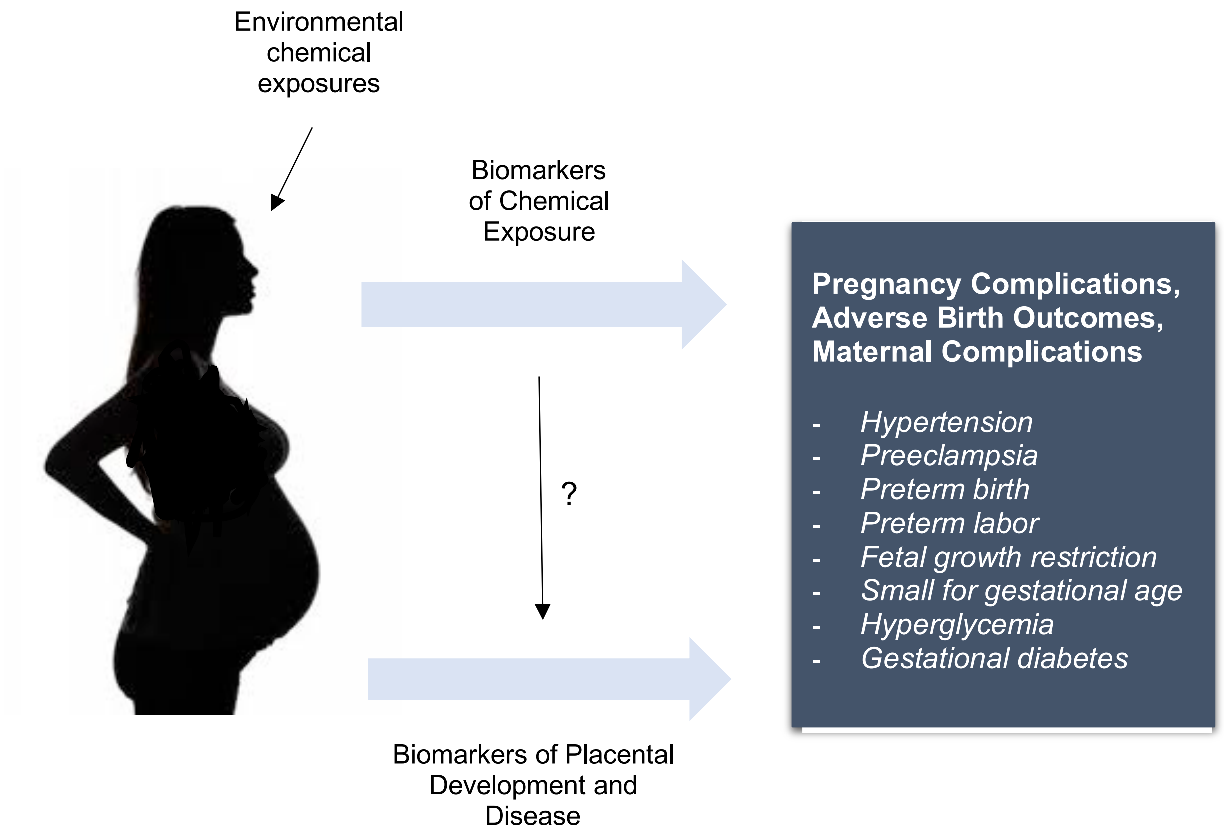

Supplemental Figure S2. Immunoreactivity of ITGA1 at the maternal-fetal interface. Representative images of floating (**A-C**) and anchoring (**D-F**) villi, interstitial invading CTBs (CTBi) of the decidua (**G-I**), and endovascular CTBs (CTBe) lining a uterine artery (**J-L**). Tissue sections of 2nd trimester samples were immunostained for ITGA1 (green) and co-stained with anti-CK (trophoblast marker, red) and DAPI (nuclear dye, blue). Images represent typical profiles of ITGA1 expression. Bars = 100 μm.

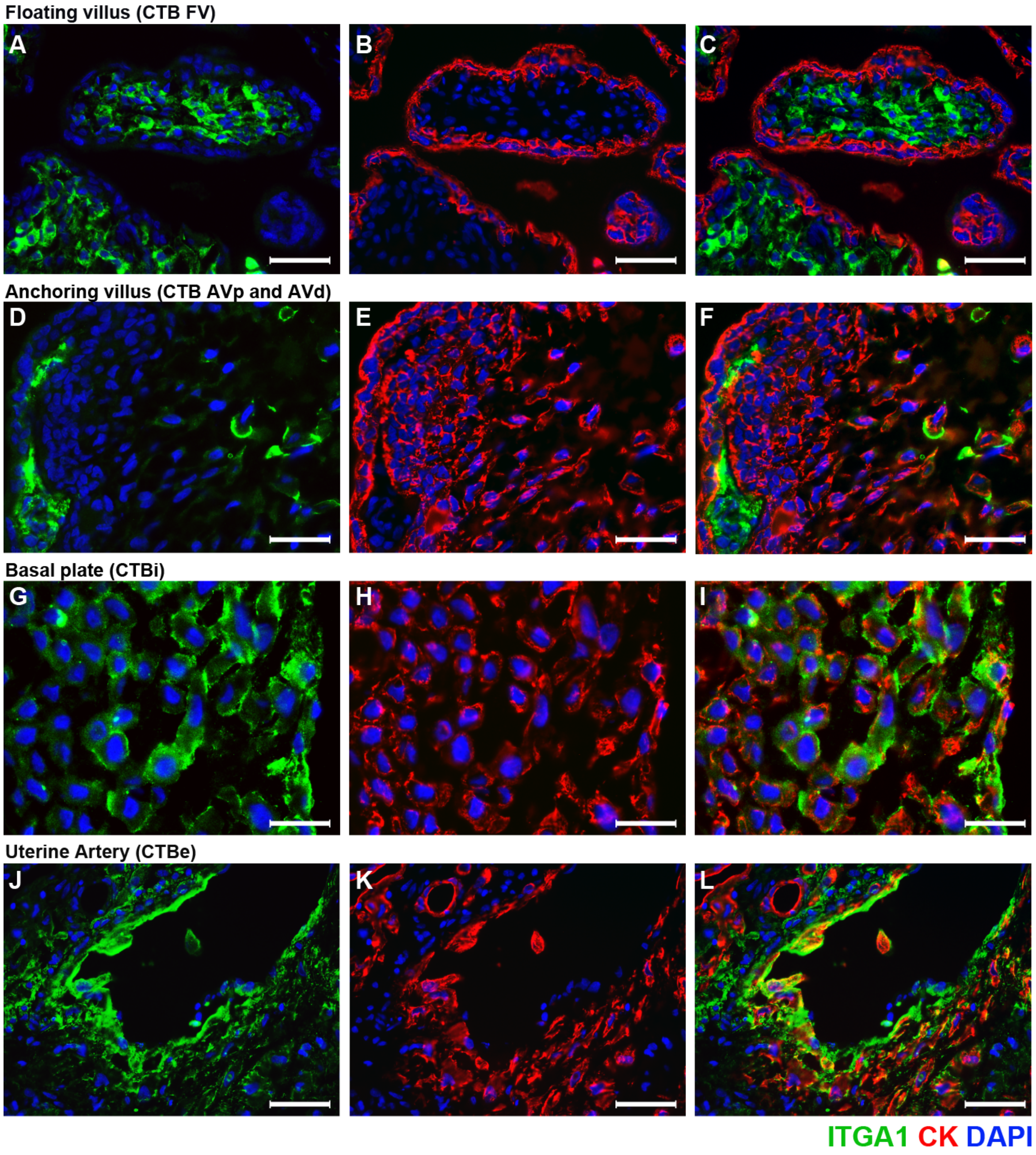

Supplemental Figure S3. Immunoreactivity of CDH5 at the maternal-fetal interface. Representative images of floating (**A-C**) and anchoring (**D-F**) villi, interstitial invading CTBs (CTBi) of the decidua (**G-I**), and endovascular CTBs (CTBe) lining a uterine artery (**J-L**). Tissue sections of 2nd trimester samples were immunostained for ITGA1 (green) and co-stained with anti-CK (trophoblast marker, red) and DAPI (nuclear dye, blue). Images represent typical profile of CDH5 expression. Bars = 100 μm.

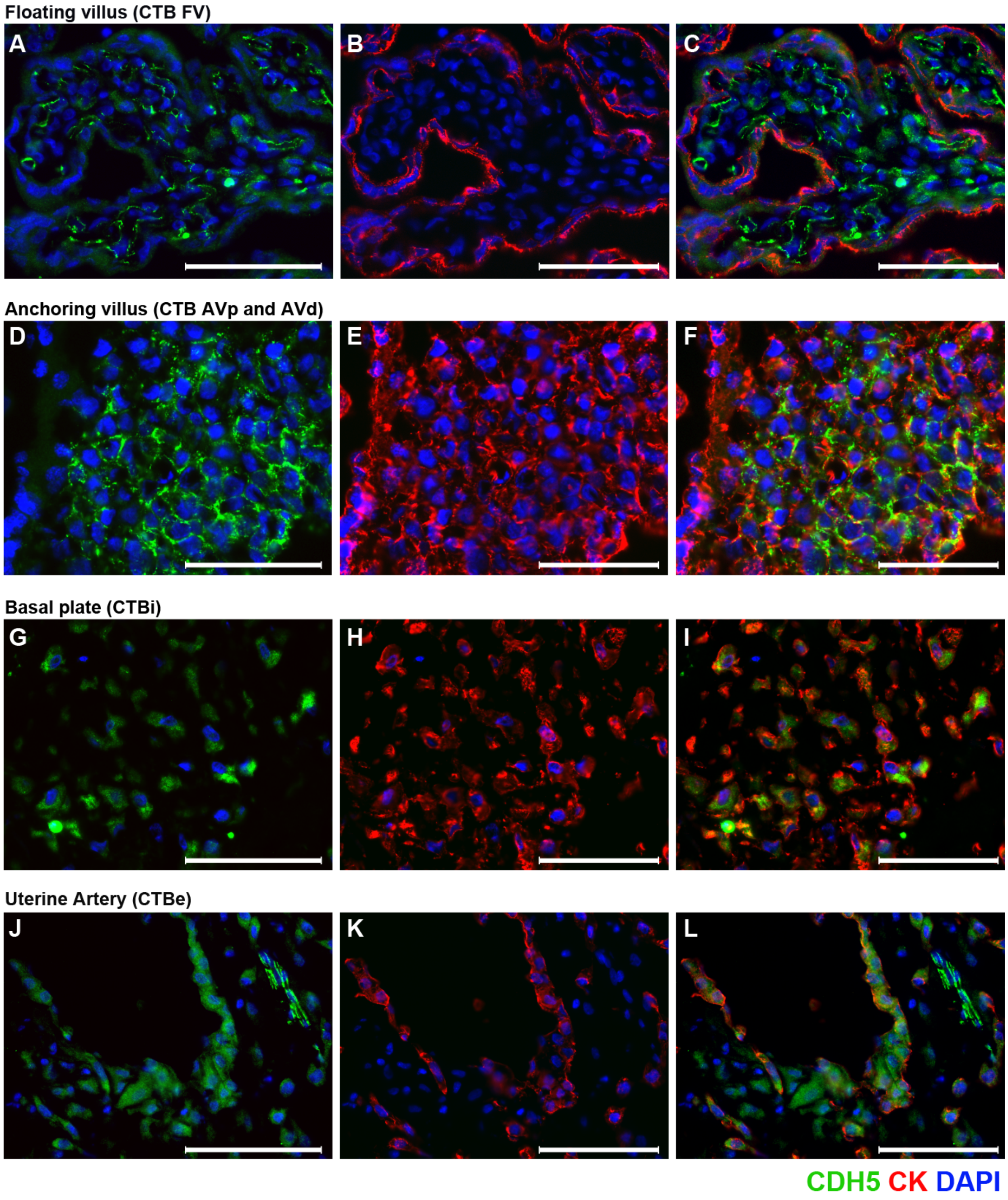

Supplemental Figure S4. Immunoreactivity of MMP1 at the maternal-fetal interface. Representative images of floating (**A-C**) and anchoring (**D-F**) villi, interstitial invading CTBs (CTBi) of the decidua (**G-I**), and endovascular CTBs (CTBe) lining a uterine artery (**J-L**). Tissue sections of 2nd trimester samples were immunostained for ITGA1 (green) and co-stained with anti-CK (trophoblast marker, red) and DAPI (nuclear dye, blue). Images represent typical profile of MMP1 expression. Bars = 100 μm.

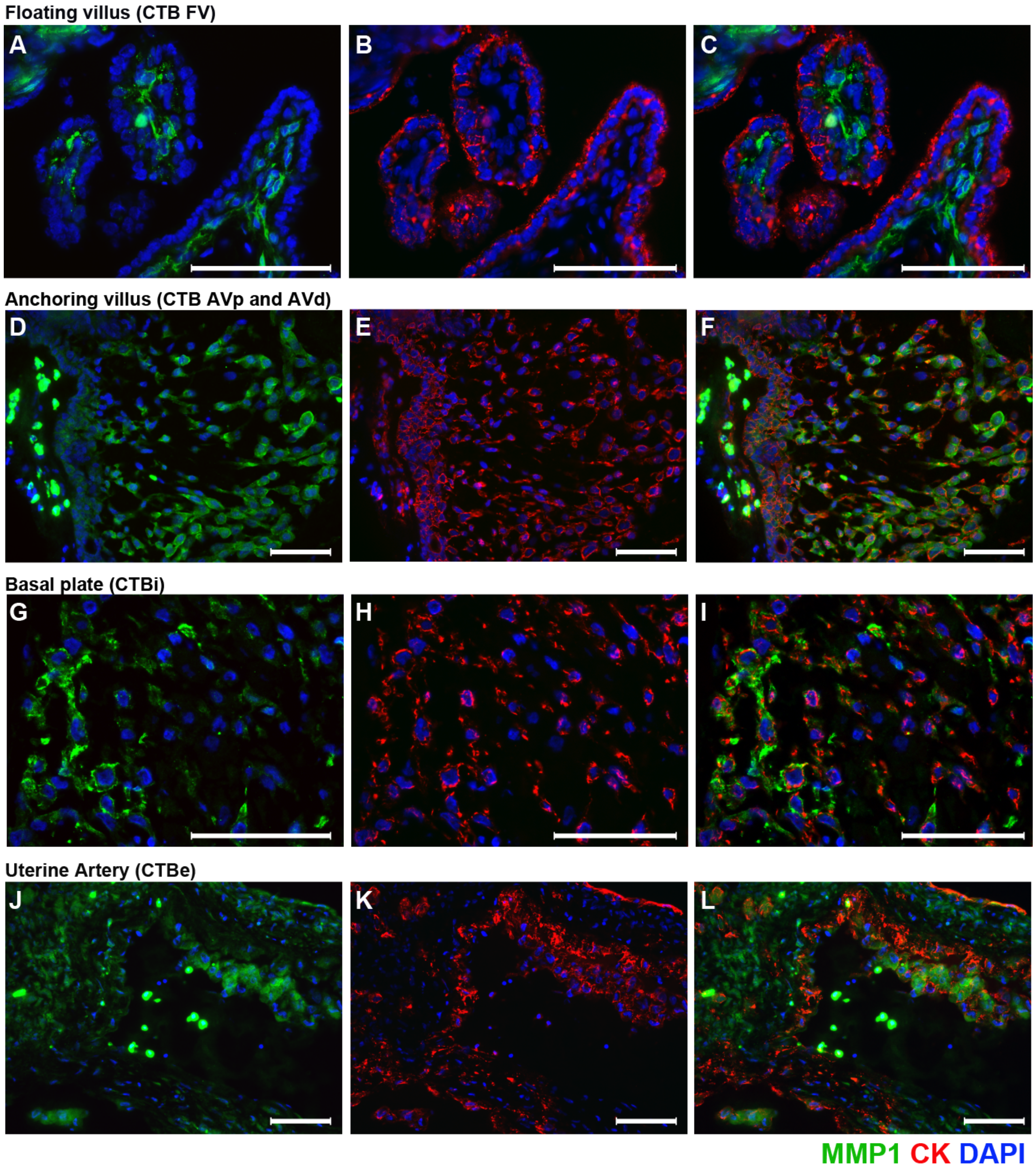

Supplemental Figure S5. Representative morphological features of the maternal-fetal interface. Representative images of morphological features that were scored in human placental biopsies: (**A**) perivillous fibrinoid deposition surrounding floating villi, (**B**) fibrinoid deposition, (**C**) leukocyte (white blood cell) infiltration of the basal plate, and (**D**) CTB remodeling of uterine arteries. Black arrows and dotted black lines indicate the feature of interest.

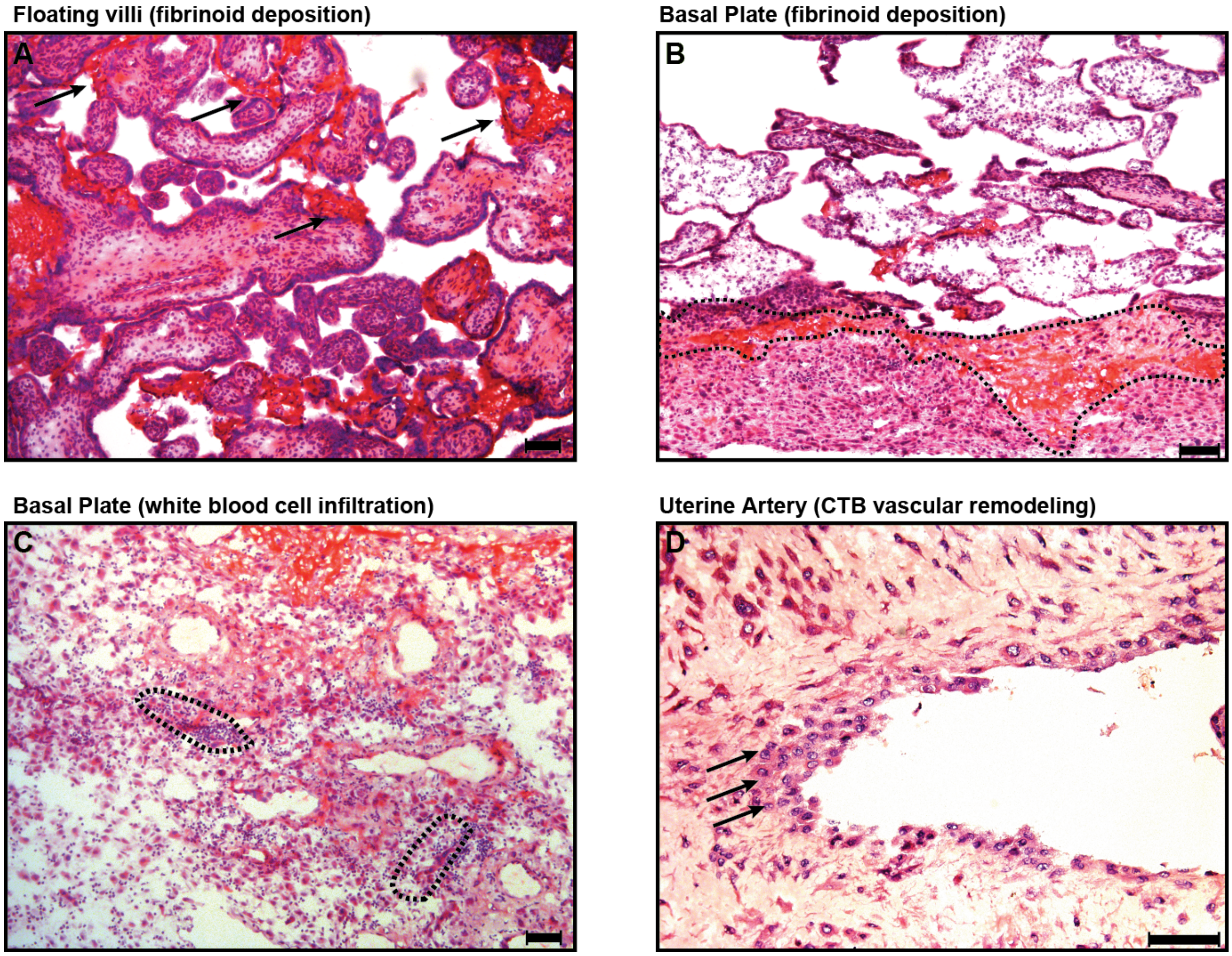

Supplement: Supplementary file 2 — Additional file 2: Figure S1. Diagram of study framework illustrating approach to evaluating relationships between biomarkers of chemical exposure and potential biomarkers of placental development and disease. Figure S2. Immunoreactivity of ITGA1 at the maternal-fetal interface. Representative images of floating (A-C) and anchoring (D-F) villi, interstitial invading CTBs (CTBi) of the decidua (G-I), and endovascular CTBs (CTBe) lining a uterine artery (J-L). Tissue sections of 2nd trimester samples were immunostained for ITGA1 (green) and co-stained with anti-CK (trophoblast marker, red) and DAPI (nuclear dye, blue). Images represent typical profiles of ITGA1 expression. Bars = 100 μm. Figure S3. Immunoreactivity of CDH5 at the maternal-fetal interface. Representative images of floating (A-C) and anchoring (D-F) villi, interstitial invading CTBs (CTBi) of the decidua (G-I), and endovascular CTBs (CTBe) lining a uterine artery (J-L). Tissue sections of 2nd trimester samples were immunostained for ITGA1 (green) and co-stained with anti-CK (trophoblast marker, red) and DAPI (nuclear dye, blue). Images represent typical profile of CDH5 expression. Bars = 100 μm. Figure S4. Immunoreactivity of MMP1 at the maternal-fetal interface. Representative images of floating (A-C) and anchoring (D-F) villi, interstitial invading CTBs (CTBi) of the decidua (G-I), and endovascular CTBs (CTBe) lining a uterine artery (J-L). Tissue sections of 2nd trimester samples were immunostained for ITGA1 (green) and co-stained with anti-CK (trophoblast marker, red) and DAPI (nuclear dye, blue). Images represent typical profile of MMP1 expression. Bars = 100 μm. Figure S5. Representative morphological features of the maternalfetal interface. Representative images of morphological features that were scored in human placental biopsies: (A) perivillous fibrinoid deposition surrounding floating villi, (B) fibrinoid deposition, (C) leukocyte (white blood cell) infiltration of the basal plate, and (D) C [file 12940_2020_617_MOESM2_ESM.pdf]
